# Authorship and citation manipulation in academic research

**DOI:** 10.1371/journal.pone.0187394

**Published:** 2017-12-06

**Authors:** Eric A. Fong, Allen W. Wilhite

**Affiliations:** 1 Department of Management, University of Alabama in Huntsville, Huntsville, Alabama, United States of America; 2 Department of Economics, University of Alabama in Huntsville, Huntsville, Alabama, United States of America; Max Planck Society, GERMANY

## Abstract

Some scholars add authors to their research papers or grant proposals even when those individuals contribute nothing to the research effort. Some journal editors coerce authors to add citations that are not pertinent to their work and some authors pad their reference lists with superfluous citations. How prevalent are these types of manipulation, why do scholars stoop to such practices, and who among us is most susceptible to such ethical lapses? This study builds a framework around how intense competition for limited journal space and research funding can encourage manipulation and then uses that framework to develop hypotheses about who manipulates and why they do so. We test those hypotheses using data from over 12,000 responses to a series of surveys sent to more than 110,000 scholars from eighteen different disciplines spread across science, engineering, social science, business, and health care. We find widespread misattribution in publications and in research proposals with significant variation by academic rank, discipline, sex, publication history, co-authors, etc. Even though the majority of scholars disapprove of such tactics, many feel pressured to make such additions while others suggest that it is just the way the game is played. The findings suggest that certain changes in the review process might help to stem this ethical decline, but progress could be slow.

## Introduction

The pressure to publish and to obtain grant funding continues to build [1–3]. In a recent survey of scholars, the number of publications was identified as the single most influential component of their performance review while the journal impact factor of their publications and order of authorship came in second and third, respectively [3]. Simultaneously, rejection rates are on the rise [4]. This combination, the pressure to increase publications coupled with the increased difficulty of publishing, can motivate academics to violate research norms [5]. Similar struggles have been identified in some disciplines in the competition for research funding [6]. For journals and the editors and publishers of those journals, impact factors have become a mark of prestige and are used by academics to determine where to submit their work, who earns tenure, and who may be awarded grants [7]. Thus, the pressure to increase a journal’s impact factor score is also increasing. With these incentives it is not surprising that academia is seeing authors and editors engaged in questionable behaviors in an attempt to increase their publication success.

There are many forms of academic misconduct that can increase an author’s chance for publication and some of the most severe cases include falsifying data, falsifying results, opportunistically interpreting statistics, and fake peer-review [5, 8–12]. For the most part, these extreme examples seem to be relatively uncommon; for example, only 1.97% of surveyed academics admit to falsifying data, although this probably understates the actual practice as these respondents report higher numbers of their colleagues misbehaving [10].

Misbehavior regarding attribution, on the other hand, seems to be widespread [13–18]; for example, in one academic study, roughly 20% of survey respondents have experienced coercive citation (when editors direct authors to add citations to articles from the editors’ journals even though there is no indicated lack of attribution and no specific articles or topics are suggested by the editor) and over 50% said they would add superfluous citations to a paper being submitted to a coercive journal in an attempt to increase its chance for publication [18]. Honorary authorship (the addition of individuals to manuscripts as authors, even though those individuals contribute little, if anything, to the actual research) is a common behavior in several disciplines [16, 17]. Some scholars pad their references in an attempt to influence journal referees or grant reviewers by citing prestigious publications or articles from the editor’s journal (or the editor’s vita) even if those citations are not pertinent to the research. While there is little systematic evidence that such a strategy influences editors, the perception of its effectiveness is enough to persuade some scholars to pad [19, 20]. Overall, it seems that many scholars consider authorship and citation to be fungible attributes, components of a project one can alter to improve their publication and funding record or to increase journal impact factors (JIFs).

Most studies examining attribution manipulation focus on the existence and extent of misconduct and typically address a narrow section of the academic universe; for example, there are numerous studies measuring the amount of honorary authorship in medicine, but few in engineering, business, or the social sciences [21–25]. And, while coercive citation has been exposed in the some business fields, less is known about its prevalence in medicine, science, or engineering. In addition, the pressure to acquire research funding is nearly as intense as publication pressures and in some disciplines funding is a major component of performance reviews. Thus, grant proposals are also viable targets of manipulation, but research into that behavior is sparse [2, 6]. However, if grant distributions are swayed by manipulation then resources are misdirected and promising areas of research could be neglected.

There is little disagreement with the sentiment that this manipulation is unethical, but there is less agreement about how to slow its use. Ultimately, to reverse this decline of ethics we need to better understand the factors that impact attribution manipulation and that is the focus of this manuscript. Using more than 12,000 responses to surveys sent to more than 110,000 academics from disciplines across the academic universe, this study aims to examine the prevalence and systematic nature of honorary authorship, coercive citation, and padded citations in eighteen different disciplines in science, engineering, medicine, business, and the social sciences. In essence, we do not just want to know how common these behaviors are, but whether there are certain types of academics who add authors or citations or are coerced more often than others. Specifically, we ask, what are the prevailing attributes of scholars who manipulate, whether willingly (e.g., padded citation) or not (e.g., coercive citation), and we consider attributes like academic rank, gender, discipline, level of co-authorship, etc. We also look into the reasons scholars manipulate and ask their opinions on the ethics of this behavior. In our opinion, a deeper understanding of manipulation can shed light on potential ways to reduce this type of academic misconduct.

## Background

As noted in the introduction, the primary component of performance reviews, and thus of individual research productivity, is the number of published articles by an academic [3]. This number depends on two things: (i) the number of manuscripts on which a scholar is listed as an author and (ii) the likelihood that each of those manuscripts will be published. The pressure to increase publications puts pressure on both of these components. In a general sense, this can be beneficial for society as it creates incentives for individuals to work harder (to increase the quantity of research projects) and to work better (to increase the quality of those projects) [6]. There are similar pressures and incentives in the application for, and distribution of, research grants as many disciplines in science, engineering, and medicine view the acquisition of funding as both a performance measure and a precursor to publication given the high expense of the equipment and supplies needed to conduct research [2, 6]. But this publication and funding pressure can also create perverse incentives.

### Honorary authorship

Working harder is not the only means of increasing an academic’s number of publications. An alternative approach is known as “honorary authorship” and it specifically refers to the inclusion of individuals as authors on manuscripts, or grant proposals, even though they did not contribute to the research effort. Numerous studies have explored the extent of honorary authorship in a variety of disciplines [17, 20, 21–25]. The motivation to add authors can come from many sources; for instance, an author may be directed to add an individual who is a department chair, lab director, or some other administrator with power, or they might voluntarily add such an individual to curry favor. Additionally, an author might create a reciprocal relationship where they add an honorary author to their own paper with the understanding that the beneficiary will return the favor on another paper in the future, or an author may just do a friend a favor and include their name on a manuscript [23, 24]. In addition, if the added author has a prestigious reputation, this can also increase the chances of the manuscript receiving a favorable review. Through these means, individuals can raise the expected value of their measured research productivity (publications) even though their actual intellectual output is unchanged.

Similar incentives apply to grant funding. Scholars who have a history of repeated funding, especially funding from the more prestigious funding agencies, are viewed favorably by their institutions [2]. Of course, grants provide resources, which increase an academic’s research output, but there are also direct benefits from funded research accruing to the university: overhead charges, equipment purchases that can be used for future projects, graduate student support, etc. Consequentially, “rainmakers” (scholars with a record of acquiring significant levels of research funding) are valued for that skill.

As with publications, the amount of research funding received by an individual depends on the number and size of proposals put forth and the probability of each getting funded. This metric creates incentives for individuals to get their names on more proposals, on bigger proposals, and to increase the likelihood that those proposals will be successful. That pressure opens the door to the same sorts of misattribution behavior found in manuscripts because honorary authorship can increase the number of grant proposals that include an author’s name and by adding a scholar with a prestigious reputation as an author they may increase their chances of being funded. As we investigate the use of honorary authorship we do not focus solely on its prevalence; we also question whether there is a systematic nature to its use. First, for example, it makes sense that academics who are early in their career have less funding and lack the protection of tenure and thus need more publications than someone with an established reputation. To begin to understand if systematic differences exist in the use of honorary authorship, the first set of empirical questions to be investigated here is: who is likely to add honorary authors to manuscripts or grant proposals? Scholars of lower rank and without tenure may be more likely to add authors, whether under pressure from senior colleagues or in their own attempt to sway reviewers. Tenure and promotion depend critically on a young scholars’ ability to establish a publication record, secure research funding, and engender support from their senior faculty. Because they lack the protection of rank and tenure, refusing to add someone could be risky. Of course, senior faculty members also have goals and aspirations that can be challenging, but junior faculty have far more on the line in terms of their career.

Second, we expect research faculty to be more likely to add honorary authors, especially to grant proposals, because they often occupy positions that are heavily dependent on a continued stream of research success, particularly regarding research funding. Third, we expect that female researchers may be less able to resist pressure to add honorary authors because women are underrepresented in faculty leadership and administrative positions in academia and lack political power [26, 27]. It is not just their own lack of position that matters; the dearth of other females as senior faculty or in leadership positions leave women with fewer mentors, senior colleagues, and administrators with similar experiences to help them navigate these political minefields [28, 29]. Fourth, because adding an author waters down the credit received by each existing author, we expect manuscripts that already have several authors to be less resistant to additional “credit sharing.” Simply put, if credit is equally distributed across authors then adding a second author would cut your perceived contribution in half, but adding a sixth author reduces your contribution by only 3% (from 20% to 17%).

Fifth, because academia is so competitive, the decisions of some scholars have an impact on others in the same research population. If your research interests are in an area in which honorary authorship is common and considered to be effective, then a promising counter-policy to the manipulation undertaken by others is to practice honorary authorship yourself. This leads us to predict that the obligation to add honorary authors to grant proposals and/or manuscripts is likely to concentrate more heavily in some disciplines. In other words, we do not expect it to be practiced uniformly or randomly across fields; instead, there will be some disciplines who are heavily engaged in adding authors and other disciplines less so engaged. In general, we have no firm predictions as to which disciplines are more likely to practice honorary authorship; we predict only that its practice will be lumpy. However, there may be reasons to suspect some patterns to emerge; for example, some disciplines, such as science, engineering, and medicine, are much more heavily dependent on research funding than other disciplines, such as the social sciences, mathematics, and business [2]. For example, over 70% of the NSF budget goes to science and engineering and about 4% to the social sciences. Similarly, most of the NIH budget goes to doctors and a smaller share to other disciplines [30]. Consequently, we suspect that the disciplines that most prominently add false investigators to grant proposals are more likely to be in science, engineering, and the medical fields. We do not expect to see that division as prominent in the addition of authors to manuscripts submitted for publication.

There are several ways scholars may internalize the pressure to perform, which can lead to different reasons why a scholar might add an honorary author to a paper. A second goal of this paper is to study who might employ these different strategies. Thus, we asked authors for the reasons they added honorary authors to their manuscripts and grants; for example, was this person in a position of authority, or a mentor, did they have a reputation that increased the chances for publication or funding, etc? Using these responses as a dependent variable, we then look to find out if these were related to the professional characteristics of the scholars in our study. The hypotheses to be tested mirror the questions posed for honorary authors. We expect junior faculty, research faculty, female faculty, and projects with more co-authors to be more likely to add additional coauthors to manuscripts and grants than professors, male faculty, and projects with fewer co-authors. Moreover, we expect for the practice to differ across disciplines. Focusing specifically on honorary authorship in grant proposals, we also explore the possibility that the use of honorary authorship differs between funding opportunities and agencies.

### Coercive citation

Journal rankings matter to editors, editorial boards, and publishers because rankings affect subscriptions and prestige. In spite of their shortcomings, impact factors have become the dominant measure of journal quality. These measures include self-citation, which creates an incentive for editors to direct authors to add citations even if those citations are irrelevant, a practice called “coercive citation” [18, 27]. This behavior has been systematically measured in business and social science disciplines [18]. Additionally, researchers have found that coercion sometimes involves more than one journal; editors have gone as far as organizing “citation cartels” where a small set of editors recommend that authors cite articles from each other’s journal [31].

When editors make decisions to coerce, who might they target, who is most likely to be coerced? Assuming editors balance the costs and benefits of their decisions, a parallel set of empirical hypotheses emerge. Returning to the various scholar attributes, we expect editors to target lower-ranked faculty members because they may have a greater incentive to cooperate as additional publications have a direct effect on their future cases for promotion, and for assistant professors on their chances of tenure as well. In addition, because they have less political clout and are less likely to openly complain about coercive treatment, lower ranked faculty members are more likely to acquiesce to the editor’s request. We predict that editors are more likely to target female scholars because female scholars hold fewer positions of authority in academia and may lack the institutional support of their male counterparts. We also expect the number of coauthors to play a role, but contrary to our honorary authorship prediction, we predict editors will target manuscripts with fewer authors rather than more authors. The rationale is simple; authors do not like to be coerced and when an editor requires additional citations on a manuscript having many authors then the editor is making a larger number of individuals aware of their coercive behavior, but coercing a sole-authored paper upsets a single individual. Notice that we are hypothesizing the opposite sign in this model than in the honorary authorship model; if *authors* are making a decision to add honorary authors then they prefer to add people to articles that already have many co-authors, but if *editors* are making the decision then they prefer to target manuscripts with few authors to minimize the potential pushback.

As was true in the model of honorary authorship, we expect the practice of coercion to be more prevalent in some disciplines than others. If one editor decides to coerce authors and if that strategy is effective, or is perceived to be effective, then there is increased pressure for other editors in the same discipline to also coerce just to maintain their ranking—if one journal climbs up in the rankings, others, who do nothing, fall. Consequently, coercion begets additional coercion and the practice can spread. But, a journal climbing up in the rankings in one discipline has little impact on other disciplines and thus we expect to find coercion practiced unevenly; prevalent in some disciplines, less so in others. Finally, as a sub-conjecture to this hypothesis, we expect coercive citation to be more prevalent in disciplines for which journal publication is the dominant measure for promotion and tenure; that is, disciplines that rely less heavily on grant funding. This means we expect the practice to be scattered, and lumpy, but we also expect relatively more coercion in the business and social sciences disciplines.

We are also interested in the types of journals that have been reported to coerce and to explore those issues we gather data using the journal as the unit of observation. As above, we expect differences between disciplines and we expect those discipline differences to mirror the discipline differences found in the author-based data set. We also expect a relationship between journal ranking and coercion because the costs and benefits of coercion differ for more or less prestigious journals. Consider the benefits of coercion. The very highest ranked journals have high impact factors; consequently, to rise another position in the rankings requires a significant increase in citations, which would require a lot of coercion. Lower-ranked journals, however, might move up several positions with relatively few coerced citations. Furthermore, consider the cost of coercion. Elite journals possess valuable reputations and risking them by coercing might be foolhardy; journals deep down in the rankings have less at stake. Given this logic, it seems likely that lower ranked journals are more likely to have practiced coercion.

We also look to see if publishers might influence the coercive decision. Journals are owned and published by many different types of organizations; the most common being commercial publishers, academic associations, and universities. *A priori*, commercial publishers, being motivated by profits, are expected to be more interested in subscriptions and sales, so the return to coercion might be higher for that group. On the other hand, the integrity of a journal might be of greater concern to non-profit academic associations and university publishers, but we don’t see a compelling reason to suppose that universities or academic associations will behave differently from one another. Finally, we control for some structural difference across journals by including each journal’s average number of cites per document and the total number of documents they publish per year.

### Padded citations

The third and final type of attribution manipulation explored here is padded reference lists. Because some editors coerce scholars to add citations to boost their journals’ impact factor score and because this practice is known by many scholars there is an incentive for scholars to add superfluous citations to their manuscripts prior to submission [18]. Provided there is an incentive for scholars to pad their reference lists in manuscripts, we wondered if grant writers would be willing to pad reference lists in grants in an attempt to influence grant reviewers.

As with honorary authorship, we suspect there may be a systematic element to padding citations. In fact, we expect the behavior of padding citations to parallel the honorary author behavior. Thus we predict that scholars of lower rank and therefore without tenure and female scholars to be more likely to pad citations to assuage an editor or sway grant reviewers. Because the practice also encompasses a feedback loop (one way to compete with scholars who pad their citations is to pad your citations) we expect the practice to proliferate in some disciplines. The number of coauthors is not expected to play a role, but we also expect knowledge of other types of manipulation to be important. That is, we hypothesize that individuals who are aware of coercion, or who have been coerced, are more likely to pad citations. With grants, we similarly expect individuals who add honorary authors to grant proposals to also be likely to pad citations in grant proposals. Essentially, the willingness to misbehave in one area is likely related to misbehavior in other areas.

## Methods

The data collection method of choice for this study is survey because to it would be difficult to determine if someone added honorary authors or padded citations prior to submission without asking that individual. As explained below, we distributed surveys in four waves over five years. Each survey, its cover email, and distribution strategy was reviewed and approved by the University of Alabama in Huntsville’s Institutional Review Board. Copies of these approvals are available on request. We purposely did not collect data that would allow us to identify individual respondents. We test our hypotheses using these survey data and journal data. Given the complexity of the data collection, both survey and archival journal data, we will begin with discussing our survey data and the variables developed from our survey. We then discuss our journal data and the variables developed there. Over the course of a five-year period and using four waves of survey collection, we sent surveys, via email, to more than 110,000 scholars in total from eighteen different disciplines (medicine, nursing, biology, chemistry, computer science, mathematics, physics, engineering, ecology, accounting, economics, finance, marketing, management, information systems, sociology, psychology, and political science) from universities across the U.S. See Table 1 for details regarding the timing of survey collection. Survey questions and raw counts of the responses to those questions are given in S1 Appendix: Statistical methods, surveys, and additional results. Complete files of all of the data used in our estimates are in the S2, S3 and S4 Appendices.

**Table 1:** Timing and coverage of surveys.

	Honorary authors: Manuscripts	Honorary Authors:Grant proposals	Padding Citations: Grant Proposals	Padding Citations: Manuscripts	Coercive citations
**Medicine**	2012	2012	2012	2012	2012
**Nursing**	2012	2012	2012	2012	2012
**Biology**	2012	2012	2012	2013	2013
**Chemistry**	2012	2012	2012	2013	2013
**Physics**	2012	2012	2012	2013	2013
**Mathematics**	2012	2012	2012	2013	2013
**Computer Science**	2012	2012	2012	2013	2013
**Engineering**	2012	2012	2012	2013	2013
**Ecology**	2012	2012	2012	2013	2013
**Accounting**	2014	2014	2014	2010	2010
**Finance**	2014	2014	2014	2010	2010
**Management**	2014	2014	2014	2010	2010
**Marketing**	2014	2014	2014	2010	2010
**Information Systems**	2014	2014	2014	2010	2010
**Economics**	2014	2014	2014	2010	2010
**Psychology**	2012	2012	2012	2010	2010
**Sociology**	2012	2012	2012	2010	2010
**Political Science**	2012	2012	2012	2010	2010

Four waves of surveys were sent to these 18 disciplines over a five year period. First wave (shaded orange) focused on coercive citation in business and the social sciences. Some of these data were used in a published study on coercive citation [18]. Second wave (pink) was early in the spring of 2012 and surveyed the health care disciplines. Third wave (green) was distributed in the fall of 2012 and asked about honorary authorship in STEM disciplines and the social sciences. The fourth wave (shaded blue) filled in the rest of the data; collecting honorary authorship data from business and coercive citation data from the sciences.

Potential survey recipients and their contact information (email addresses) were identified in three different ways. First, we were able to get contact information for management scholars through the Academy Management using the annual meeting catalog. Second, for economics and physicians we used the membership services provided by the American Economic Association and the American Medical Association. Third, for the remaining disciplines we identified the top 200 universities in the United States using *U*.*S*. *News and World Report’s* “National University Rankings” and hand-collected email addresses by visiting those university websites and copying contact information for individual faculty members from each of the disciplines. We also augmented the physician contact list by visiting the web sites of the medical schools in these top 200 school as well. With each wave of surveys, we sent at least one reminder to participate. The approximately 110,000 surveys yielded about 12,000 responses for an overall response rate of about 10.5%. Response rates by discipline can be found in Table A in S1 Appendix.

Few studies have examined the systematic nature of honorary authorship and padded citation and thus we developed our own survey items to address our hypotheses. Our survey items for coercive citation were taken from prior research on coercion [18]. All survey items and the response alternatives with raw data counts are given in S1 Appendix. The complete data are made available in S2–S4 Appendices.

Our first set of tests relate to honorary authorship in manuscripts and grants and is made up of several dependent variables, each related to the research question being addressed. We begin with the existence of honorary authorship in manuscripts. This dependent variable is composed of the answers to the survey question: “Have YOU felt obligated to add the name of another individual as a coauthor to your manuscript even though that individual’s contribution was minimal?” Responses were in the form of yes and no where “yes” was coded as a 1 and “no” coded as a 0. The next dependent variable addresses the frequency of this behavior asking: “In the last five years HOW MANY TIMES have you added or had coauthors added to your manuscripts even though they contributed little to the study?” The final honorary authorship dependent variables deal with the reason for including an honorary author in manuscripts: “Even though this individual added little to this manuscript he (or she) was included as an author. The main reason for this inclusion was:” and the choices regarding this answer were that the honorary author is the director of the lab or facility used in the research, occupies a position of authority and can influence my career, is my mentor, is a colleague I wanted to help out, was included for reciprocity (I was included or expect to be included as a co-author on their work), has data I needed, has a reputation that increases the chances of the work being published, or they had funding we could apply to the research. Responses were coded as 1 for the main reason given (only one reason could be selected as the “main” reason) and 0 otherwise.

Regarding honorary authorship in grant proposals, our first dependent variable addresses its existence: “Have you ever felt obligated to add a scholar’s name to a grant proposal even though you knew that individual would not make a significant contribution to the research effort?” Again, responses were in the form of yes and no where “yes” was coded as a 1 and “no” coded as a 0. The remaining dependent variables regarding honorary authorship in grant proposals addresses the reasons for adding honorary authors to proposals: “The main reason you added an individual to this grant proposal even though he (or she) was not expected to make a significant contribution was:” and the provided potential responses were that the honorary author is the director of the lab or facility used in the research, occupies a position of authority and can influence my career, is my mentor, is a colleague I wanted to help out, was included for reciprocity (I was included or expect to be included as a co-author on their work), has data I needed, has a reputation that increases the chances of the work being published, or was a person suggested by the grant reviewers. Responses were coded as 1 for the main reason given (only one reason could be selected as the “main” reason) and 0 otherwise.

Our next major set of dependent variables deal with coercive citation. The first coercive citation dependent variable was measured using the survey question: “Have YOU received a request from an editor to add citations from the editor’s journal for reasons that were not based on content?” Responses were in the form of yes (coded as a 1) and no (coded as 0). The next question deals with the frequency: “In the last five years, approximately HOW MANY TIMES have you received a request from the editor to add more citations from the editor’s journal for reasons that were not based on content?”

Our final set of dependent variables from our survey data investigates padding citations in manuscripts and grants. The dependent variable that addresses an author’s willingness to pad citations for manuscripts comes from the following question: “If I were submitting an article to a journal with a reputation of asking for citations to itself even if those citations are not critical to the content of the article, I would probably add such citations BEFORE SUBMISSION.” Answers to this question were in the form of a Likert scale with five potential responses (Strongly Disagree, Disagree, Neutral, Agree, and Strongly Agree) where Strongly Disagree was coded as a 1 and Strongly Agree coded as a 5. The dependent variable for padding citations in grant proposals uses responses to the statement: “When developing a grant proposal I tend to skew my citations toward high impact factor journals, even if those citations are of marginal import to my proposal.” Answers were in the form of a Likert scale with five potential responses (Strongly Disagree, Disagree, Neutral, Agree, and Strongly Agree) where Strongly Disagree was coded as a 1 and Strongly Agree coded as a 5.

To test our research questions, several independent variables were developed. We begin by addressing the independent variables that cut across honorary authorship, coercive citation, and padding citations. The first is academic rank. We asked respondents their current rank: Assistant Professor, Associate Professor, Professor, Research Faculty, Clinical Faculty, and other. Dummy variables were created for each category with Professor being the omitted category in our tests of the hypotheses. The second general independent variable is discipline: Medicine, Nursing, Accounting, Economics, Finance, Information Systems, Management, Marketing, Political Science, Psychology, Sociology, Biology, Chemistry, Computer Science, Ecology, and Engineering. Again, dummy variables were created for each discipline, but instead of omitting a reference category we include all disciplines and then constrain the sum of their coefficients to equal zero. With this approach, the estimated coefficients then tell us how each discipline differs from the average level of honorary authorship, coercive citation, or padded citation across the academic spectrum [32]. We can conveniently identify three categories: (i) disciplines that are significantly more likely to engage in honorary authorship, coercive citation, or padded citation than the average across all disciplines, (ii) disciplines that do not differ significantly from the average level of honorary authorship, coercive citation, or padded citation across all of these disciplines, and (iii) those who are significantly less likely to engage in honorary authorship, coercive citation, or padded citation than the average. We test the potential gender differences with a dummy variable male = 1, females = 0.

Additional independent variables were developed for specific research questions. In our tests of honorary authorship, there is an independent variable addressing the number of co-authors on a respondent’s most recent manuscript. If the respondent stated that they have added an honorary author then they were asked “Please focus on the most recent incidence in which an individual was added as a coauthor to one of your manuscripts even though his or her contribution was minimal. Including yourself, how many authors were on this manuscript?” Respondents who had not added an honorary author were asked to report the number of authors on their most recently accepted manuscript. We also include an independent variable regarding funding agencies: “To which agency, organization, or foundation was this proposal directed?” Again, for those who have added authors, we request they focus on the most recent proposal where they used honorary authorship and for those who responded that they have not practiced honorary authorship, we asked where they sent their most recent proposal. Their responses include NSF, HHS, Corporations, Private nonprofit, State funding, Other Federal grants, and Other grants. Regarding coercive citation, we included an independent variable regarding number of co-authors on their most recent coercive experience and thus if a respondent indicated they’ve been coerced we asked: “Please focus on the most recent incident in which an editor asked you to add citations not based on content. Including yourself, how many authors were on this manuscript?” If a respondent indicated they’ve never been coerced, we asked them to state the number of authors on their most recently accepted manuscript.

Finally, we included control variables. In our tests, we included the respondent’s performance or exposure to these behaviors. For those analyses focusing on manuscripts we used acceptances: “Within the last five years, approximately how many publications, including acceptances, do you have?” The more someone publishes, the more opportunities they have to be coerced, add authors, or add citations; thus, scholars who have published more articles are more likely to have experienced coercion, ceteris paribus. And in our tests of grants we used two performance indicators: 1) “In the last five years approximately how many grant proposals have you submitted for funding?” and 2) “Approximately how much grant money have you received in the last five years? Please write your estimated dollars in box; enter 0 if zero.”

We also investigate coercion using a journal-based dataset, Scopus, which contains information on more than 16,000 journals from these 18 disciplines [33]. It includes information on the number of articles published each year, the average number of citations per manuscript, the rank of the journal, disciplines that most frequently publish in the journal, the publisher, and so forth. These data were used to help develop our dependent variable as well as our independent and control variables for the journal analysis. Our raw journal data is provided in S4 Appendix: Journal data.

The dependent variables in our journal analysis measure whether a specific journal was identified as a journal in which coercion occurred, or not, and the frequency of that identification. Survey respondents were asked: “To track the possible spread of this practice we need to know specific journals. Would you please provide the names of journals you know engage in this practice?” Respondents were given a blank space to write in journal names. The majority of our respondents declined to identify journals where coercion has occurred; however, more than 1200 respondents provided journal names and in some instances, respondents provided more than one journal name. Among the population of journals in the Scopus database, 612 of these were identified as journals that have coerced by our survey respondents, some of these journals were identified several times. The first dependent variable is binary, coded as 1 if a journal was identified as a journal that has coerced, and coded as 0 otherwise. The frequency estimates uses the count, how many times they were named, as the dependent variable.

The independent variables measure various journal attributes, the first being discipline. The Scopus database identifies the discipline that most frequently publishes in any given journal, and that information was used to classify journals by discipline. Thus, if physics is the most common discipline to publish in a journal, it was classified as a physics journal. We look to see if there is a publisher effect using the publisher information in Scopus to create four categories: commercial publishers, academic associations, universities, and others (the omitted reference category).

We also control for differing editorial norms across disciplines. First, we include the number of documents published annually by each journal. All else equal, a journal that publishes more articles has more opportunities to engage in coercion, and/or it interacts with more authors and is more likely to be reported in our sample. Second, we control for the average number of citations per article. The average number of citations per document controls for some of the overall differences in citation practices across disciplines.

Given the large number of hypotheses to be tested, we present a compiled list of the dependent variables in Table 2. This table names the dependent variables, describes how they were constructed, and lists the tables that present the estimated coefficients pertinent to those dependent variables. Table 2 is intended to give readers an outline of the arc of the remainder of the manuscript.

**Table 2:** List of dependent variables, a description of how those variables are constructed, and the table in which they appear.

Dependent variable	Description	Table
***Honorary Authorship*: *Manuscripts***		
**Added honorary author to manuscript**	Binary variable = 1 if respondent has added an honorary author to a research manuscript in the last five years; = 0 otherwise	Table 3
**Number of times added authors to manuscripts**	Count variable; number of times have added honorary author to manuscripts in the last five years	Table 4
***Honorary Authorship*: *Grant Proposals***		
**Added honorary author to grant proposal**	Binary variable = 1 if respondent has added an honorary author to a grant proposal in the last five years; = 0 otherwise	Table 5
***Reasons added Honorary Authors to Manuscripts***		
**Director**	Binary variable = 1 the primary reason this honorary author was added to a manuscript; “was the Director of the lab or facility used in the research.” = 0 otherwise	Table 6
**Authority**	Binary variable = 1 the primary reason this honorary author was added to a manuscript; “occupies a position of authority and can influence my career.” = 0 otherwise.	Table 6
**Mentor**	Binary variable = 1 the primary reason this honorary author was added to a manuscript, “this is my mentor.” = 0 otherwise	Table 6
***Reasons added Honorary Authors to Grant Proposals***		
**Reputation**	Binary variable = 1 the primary reason this honorary author was added to a grant proposal, “their reputation increases the chances of receiving funding.” = 0 otherwise	Table 7
**Director**	Binary variable = 1 the primary reason this honorary author was added to a grant proposal, “was the Director of the lab or facility used in the research.” = 0 otherwise	Table 7
**Authority**	Binary variable = 1 the primary reason this honorary author was added to a grant proposal, this individual, “occupies a position of authority and can influence my career.” = 0 otherwise	Table 7
***Coercive Citations*: *individual data***		
**Existence of coercive citation**	Binary variable = 1 if respondent was coerced by an editor to add superfluous citations to the editor’s journal in the last five years. = 0 otherwise	Table 8
**Frequency of coercive citation**	Count variable; number of times respondent was coerced by editors to add superfluous citations to the editors’ journals in the last five years.	Table 9
***Coercive Citations*: *journal data***		
**Journals that have coerced**	Binary data = 1 if journal was named as having coerced; = 0 otherwise	Tables 10 and 11
**Frequency journals coerced authors**	Count variable; number of times a journal was identified as one that practiced coercion in the last five years	Tables 10 and 11
***Padded Citations***		
**Padded citations in manuscripts**	Ordered categorical variable; Response to the statement, “If I were submitting an article to a journal with a reputation of asking for citations to itself even if those citations are not critical to the content of the article, I would probably add such citations BEFORE SUBMISSION.” Strongly agree = 5; agree = 4; neutral = 3; disagree = 2; strongly disagree = 1	Table 12
**Padded citations in grant proposals**	Ordered categorical variable; response to the statement, “When developing a grant proposal I tend to skew my citations toward high impact factor journals even if those citations are of marginal impact to my proposal.” Strongly agree = 5; agree = 4; neutral = 3; disagree = 2; strongly disagree = 1	Table 13

## Results

### Honorary authorship in research manuscripts

**Fig 1:**
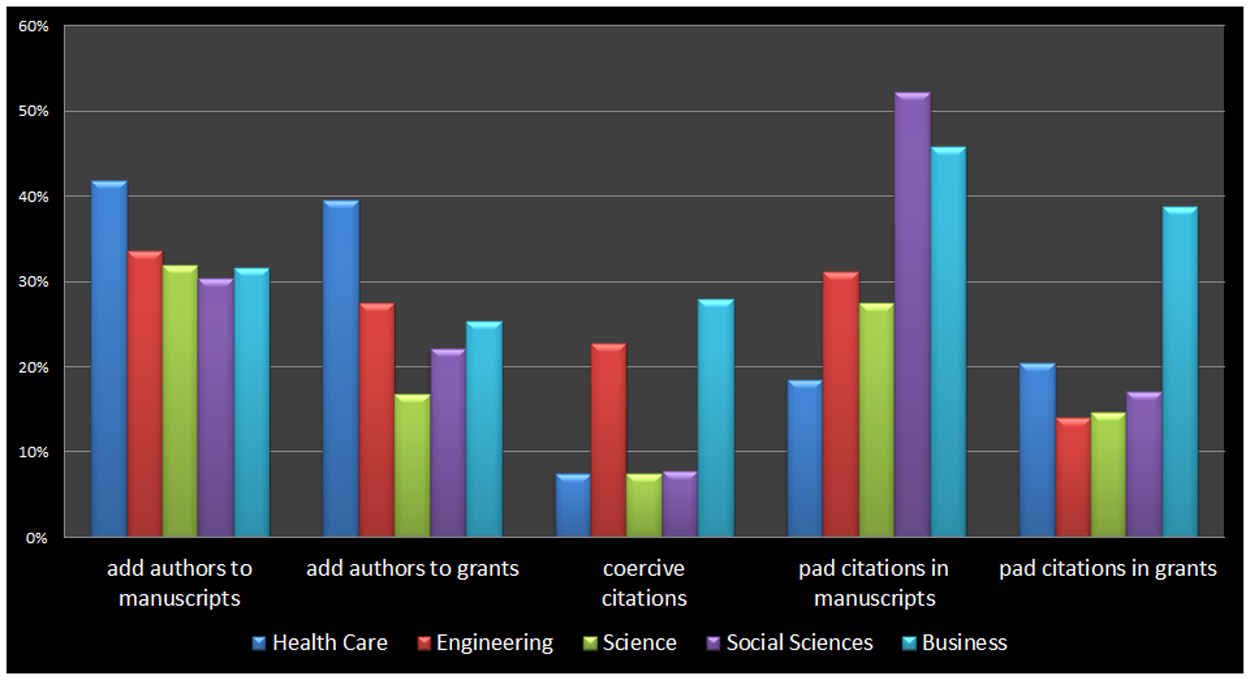
Manipulation of authorship and citation across academia. Percentage of respondents who report that honorary authors have been added to their research projects, they have been coerced by editor to add citations, or who have padded their citations, sorted by field of study and type of manipulation.

Looking across all disciplines, 35.5% of our survey respondents report that they have added an author to a manuscript even though the contribution of those authors was minimal. Fig 1 displays tallies of some raw responses to show how the use of honorary authorship, for both manuscripts and grants, differs across science, engineering, medicine, business, and the social sciences.

To begin the empirical study of the systematic use of honorary authorship, we start with the addition of honorary authors to research manuscripts. This is a logit model in which the dependent variable equals one if the respondent felt obligated to add an author to their manuscript, “even though that individual’s contribution was minimal.” The estimates appear in Table 3. In brief, all of our conjectures are observed in these data. As we hypothesized above, the pressure on scholars to add authors “who do not add substantially to the research project,” is more likely to be felt by assistant professors and associate professors relative to professors (the reference category). To understand the size of the effect, we calculate odds ratios (*e*^*β*^) for each variable, also reported in Table 3. Relative to a full professor, being an assistant professor increases the odds of honorary authorship in manuscripts by 90%, being an associate professor increases those odds by 40%, and research faculty are twice as likely as a professor to add an honorary author.

**Table 3:** Adding honorary authors to manuscripts: Estimate coefficients and odds ratios.

Variables	Estimated coefficients	Std. error	Odds ratio	Std. error
***Academic Ranks***				
**Assistant Professor**	0.642**	0.061	1.901**	0.117
**Associate Professor**	0.341**	0.056	1.407**	0.079
**Lecturer**	0.085	0.137	1.089	0.150
**Research Faculty**	0.716**	0.129	2.046**	0.265
**Clinical Faculty**	0.411*	0.169	1.508*	0.255
**Other rank**	0.504**	0.127	1.655**	0.211
***Gender and number of co-authors***				
**Male**	-0.471**	0.050	0.624**	0.031
**# co-authors**	0.034**	0.008	1.035**	0.009
***Disciplines***				
**Medicine**	0.191**	0.055	1.211**	0.068
**Nursing**	0.148	0.084	1.161	0.098
**Accounting**	-0.615**	0.200	0.541**	0.108
**Economics**	-0.218*	0.094	0.804*	0.075
**Finance**	-0.105	0.195	0.900	0.175
**Info systems**	0.377	0.209	1.458*	0.305
**Management**	0.491**	0.089	1.634**	0.146
**Marketing**	0.561**	0.149	1.752**	0.262
**Political Science**	-0.819**	0.141	0.441**	0.062
**Psychology**	0.056	0.076	1.058	0.080
**Sociology**	0.052	0.101	1.054	0.107
**Biology**	0.123	0.068	1.131	0.077
**Chemistry**	-0.352**	0.103	0.703**	0.073
**Computer Sci**	0.040	0.131	1.041	0.136
**Ecology**	0.300**	0.113	1.349**	0.153
**Engineer**	0.145	0.088	1.156	0.101
**Mathematics**	-0.527**	0.170	0.590**	0.100
**Physics**	0.151	0.110	1.163	0.128
***Publication history***				
**Publications**	0.014**	0.002	1.014**	0.002
**Constant**	-0.986**	0.063	0.373**	0.023
	n = 9910; χ^2^ = 524.11			

Logit regression, dependent variable is binary: 1 = felt obligated to add an author, 0 = did not feel obligated. Independent variables include academic ranks, disciplines, gender, number of co-authors, and the number of publications. Discipline estimates compare each discipline to the overall average across all disciplines.

* Indicates significance at the 5% level;

** significant at the 1% level.

Consistent with our hypothesis, we found support that females were more likely to add honorary authors as the estimated coefficient on males was negative and statistically significant. The odds that a male feels obligated to add an author to a manuscript is 38% lower than for females. As hypothesized, authors who already have several co-authors on a manuscript seem more willing to add another; consistent with our hypotheses that the decrement in individual credit diminishes as the number of authors rises. Overall, these results align with our fundamental thesis that authors are purposively deciding to deceive, adding authors when the benefits are higher and the costs lower.

Considering the addition of honorary authors to manuscripts, Table 3 shows that four disciplines are statistically more likely to add honorary authors than the average across all disciplines. Listing those disciplines in order of their odds ratios and starting with the greatest odds, they are: marketing, management, ecology, and medicine (physicians). There are five disciplines in which honorary authorship is statistically below the average and starting with the lowest odds ratio they are: political science, accounting, mathematics, chemistry, and economics. Finally, the remaining disciplines, statistically indistinguishable from the average, are: physics, psychology, sociology, computer science, finance, engineering, biology, information systems, and nursing. At the extremes, scholars in marketing are 75% more likely to feel an obligation to add authors to a manuscript than the average across all disciplines while political scientists are 44% less likely than the average to add an honorary author to a manuscript.

To bolster these results, we also asked individuals to tell us *how many times* they felt obligated to add honorary authors to manuscripts in the last five years. Using these responses as our dependent variable we estimated a negative binomial regression equation with the same independent variables used in Table 3. The estimated coefficients and their transformation into incidence rate ratios are given in Table 4. Most of the estimated coefficients in Tables 3 and 4 have the same sign and, with minor differences, similar significance levels, which suggests the attributes associated with a higher likelihood of adding authors are also related to the frequency of that activity. Looking at the incidence rate ratios in Table 4, scholars occupying the lower academic ranks, research professors, females, and manuscripts that already have many authors more frequently add authors. Table 4 also suggests that three additional disciplines, Nursing, Biology, and Engineering, have more incidents of adding honorary authors to manuscripts than the average of all disciplines and, consequently, the disciplines that most frequently engage in honorary authorship are, by effect size, management, marketing, ecology, engineering, nursing, biology, and medicine.

**Table 4:** Number of times authors added to manuscripts: Estimated coefficients and incidence rate ratios.

	Estimated coefficient	Standard Error	Incidence rate ratio	Standard error
***Faculty Ranks***				
**Assistant Professor**	0.658**	0.059	1.931**	0.113
**Associate Professor**	0.343**	0.054	1.409**	0.076
**Lecturer**	0.147	0.135	1.159	0.157
**Research Faculty**	0.801**	0.123	2.227**	0.274
**Clinical Fac.**	0.175	0.173	1.192	0.206
**Other rank**	0.501**	0.122	1.650**	0.201
***Gender and number of co-authors***				
**Male**	-0.266**	0.049	0.766**	0.037
**Number of co-authors**	0.084**	0.010	1.088**	0.010
***Disciplines***				
**Medicine**	0.138**	0.054	1.148**	0.062
**Nursing**	0.201*	0.083	1.223**	0.102
**Accounting**	-0.650**	0.199	0.552**	0.104
**Economics**	-0.072	0.089	0.930	0.083
**Finance**	-0.070	0.189	0.932	0.176
**Info systems**	0.254	0.209	1.289	0.269
**Management**	0.515**	0.087	1.674**	0.146
**Marketing**	0.398**	0.150	1.488**	0.222
**Political Science**	-0.718**	0.134	0.487**	0.065
**Psychology**	0.044	0.074	0.957	0.071
**Sociology**	0.010	0.101	0.990	0.100
**Biology**	0.149*	0.066	1.161*	0.076
**Chemistry**	-0.587**	0.104	0.555**	0.058
**Computer Science**	0.111	0.126	1.118	0.141
**Ecology**	0.325**	0.109	1.383**	0.150
**Engineering**	0.299**	0.083	1.348**	0.112
**Mathematics**	-0.317*	0.154	0.728*	0.112
**Physics**	0.078	0.107	1.081	0.115
***Other controls***				
**Publications**	0.025**	0.002	1.025**	0.002
**Constant**	-1.086**	0.063	0.337**	0.021
	n = 9929; χ^2^ = 731.5			

Negative binomial regression, the dependent variable is the number of times the respondent added honorary authors to manuscripts in the last five years. Independent variables include academic ranks, disciplines, gender, number of co-authors, and the number of publications. Discipline estimates compare each discipline to the overall average across all disciplines.

* Indicates significance at the 5% level;

** significant at the 1% level.

Another way to measure effect sizes is to standardize the variables so that the changes in the odds ratios or incidence rate ratios measure the impact of a one standard deviation change of the independent variable on the dependent variable. In Tables 3 and 4, the continuous variables are the number of coauthors on the particular manuscripts of interest and the number of publications of each respondent. Tables C and D (in S1 Appendix) show the estimated coefficients and odds ratios with standardized coefficients. Comparing the two sets of results is instructive. In Table 3, the odds ratio for the number of coauthors is 1.035, adding each additional author increases the odds of this manuscript having an honorary author by 3.5%. The estimated odds ratio for the standardized coefficient, (Table C in S1 Appendix) is 1.10, meaning an increase in the number of coauthors of one standard deviation increases the odds that this manuscript has an honorary author by 10%. Meanwhile the standard deviation of the number of coauthors in this sample is 2.78, so 3.5% x 2.78 = 9.73%; the two estimates are very similar. This similarity repeats itself when we consider the number of publications and when we compare the incidence rate ratios across Table 4 and Table D in S1 Appendix. Standardization also tells us something about the relative effect size of different independent variables and in both models a standard deviation increase in the number of coauthors has a larger impact on the likelihood of adding another author than a standard deviation increase in additional publications.

### Honorary authorship in grant proposals

Our next set of results focus on honorary authorship in grant proposals. Looking across all disciplines, 20.8% of the respondents reported that they had added an investigator to a grant proposal even though the contribution of that individual was minimal (see Fig 1 for differences across disciplines). To more deeply probe into that behavior we begin with a model in which the dependent variable is binary, whether a respondent has added an honorary author, or not, to a grant proposal and thus use a logit model. With some modifications, the independent variables include the same variables as the manuscript models in Tables 3 and 4. We remove a control variable relevant to manuscripts (total number of publications) and add two control variables to measure the level of exposure a particular scholar has to the funding process: the number of grants funded in the last five years and the total amount of grant funding (dollars) in that same period.

The results appear in Table 5 and, again, we see significant participation in honorary authorship. The estimates largely follow our predictions and mirror the results of the models in Tables 3 and 4. Academic rank has a smaller effect, being an assistant professor increases the odds of adding an honorary author to a grant by 68% and being an associate professor increases those odds by 52%. On the other hand, the impact of being a research professor is larger in the grant proposal models than the manuscripts model of Table 3 while the impact of sex is smaller. As was true in the manuscripts models, the obligation to add honorary authors is also lumpy, some disciplines being much more likely to engage in the practice than others. We find five disciplines in the “more likely than average” category: medicine, nursing, management, engineering, and psychology. The disciplines that tend to add fewer honorary authors to grants are political science, biology, chemistry, and physics. Those that are indistinguishable from the average are accounting, economics, finance, information systems, sociology, ecology, marketing, computer science, and mathematics.

**Table 5:** Adding honorary authors to grant proposals: Estimated coefficients and odds ratios.

Variables	Estimated coefficients	Std. error	Odds ratio	Std. error
***Faculty Ranks***				
**Assistant Professor**	0.523**	0.076	1.687**	0.128
**Associate Professor**	0.424**	0.071	1.528**	0.108
**Lecturer**	0.814**	0.203	2.258**	0.459
**Research Faculty**	0.966**	0.148	2.628**	0.388
**Clinical Faculty**	0.004	0.247	1.004	0.248
**Other rank**	0.799**	0.182	2.223**	0.405
***Disciplines***				
**Medicine**	0.792**	0.068	2.208**	0.151
**Nursing**	0.786**	0.104	2.195**	0.229
**Accounting**	0.139	0.280	1.149	0.322
**Economics**	0.158	0.128	1.171	0.150
**Finance**	0.014	0.336	1.014	0.341
**Info systems**	-0.032	0.335	0.968	0.325
**Management**	0.306**	0.131	1.358**	0.178
**Marketing**	-0.077	0.283	0.926	0.262
**Political Sci**	-0.729**	0.202	0.482**	0.097
**Psychology**	0.234*	0.097	1.264**	0.123
**Sociology**	-0.013	0.140	0.987	0.138
**Biology**	-0.453**	0.099	0.636**	0.063
**Chemistry**	-0.385**	0.138	0.680**	0.094
**Computer Sci**	0.061	0.171	1.063	0.181
**Ecology**	-0.264	0.154	0.768	0.118
**Engineer**	0.238*	0.110	1.269*	0.140
**Mathematics**	-0.457	0.243	0.633	0.154
**Physics**	-0.317*	0.155	0.728*	0.113
***Gender and other control variables***				
**Male**	-0.252**	0.063	0.777**	0.049
**# grants**	0.032**	0.004	1.032	0.004
**Grant dollars**	-2.40E-10	2.8E-10	1.00	2.8E-10
**Constant**	-1.710**	0.079	0.181**	0.014
n = 7524; χ^2^ = 437.01				

Logit regression, dependent variable is binary: 1 = added author, 0 = did not add author to research. Independent variables include academic ranks, disciplines, gender, number of grants received, and total grant money received in last 5 years.

* Indicates significance at the 5% level;

** significant at the 1% level.

We speculated that science, engineering, and medicine were more likely to practice honorary authorship in grant proposals because those disciplines are more dependent on research funding and more likely to consider funding as a requirement for tenure and promotion. The results in Tables 3 and 5 are somewhat consistent with this conjecture. Of the five disciplines in the “above average” category for adding honorary authors to grant proposals, four (medicine, nursing, engineering, and psychology) are dependent on labs and funding to build and maintain such labs for their research.

### Reasons for adding honorary authors

**Fig 2:**
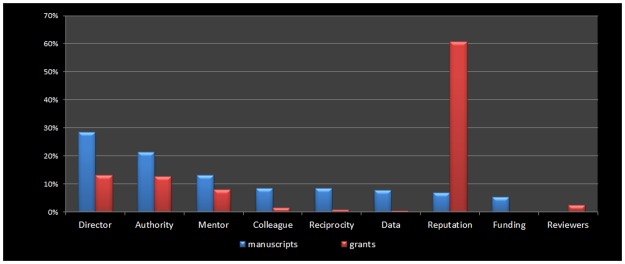
Reasons for adding honorary authors to grants and manuscripts. Each pair of columns presents the percentage of responses who selected a particular reason for adding an honorary author to a manuscript or a grant proposal. Director refers to responses stating, “this individual was the director of the lab or facility used in the research.” Authority refers to responses stating, “this individual occupies a position of authority and can influence my career.” Mentor, “this is my mentor”; colleague, “this a colleague I wanted to help”; reciprocity, “I was included or expect to be included as a co-author on their work”; data, “they had data I needed”; reputation, “their reputation increases the chances of the work being published (or funded)”; funding, “they had funding we could apply to the research”; and reviewers, “the grant reviewers suggested we add co-authors.”

Our next set of results looks more deeply into the reasons scholars give for adding honorary authors to manuscripts and to grants. When considering honorary authors added to manuscripts, we focus on a set of responses to the question: “what was the major reason you felt you needed to add those co-author(s)?” When we look at grant proposals, we use responses to the survey question: “The main reason you added an individual to this grant proposal even though he (or she) was not expected to make a significant contribution was…” Starting with manuscripts, although nine different reasons for adding authors were cited (see survey in S1 Appendix), only three were cited more than 10% of the time. The most common reason our respondents added honorary authors (28.4% of these responses) was because the added individual was the director of the lab. The second most common reason (21.4% of these responses), and the most disturbing, was that the added individual was in a position of authority and could affect the scholar’s career. Third among the reasons for honorary authorship (13.2%) were mentors. “Other” was selected by about 13% of respondents. The percentage of raw responses for each reason is shown in Fig 2.

To find out if the three most common responses were related to the professional characteristics of the scholars in our study, we re-estimate the model in Table 3 after replacing the dependent variable with the reasons for adding an author. In other words, the first model displayed in Table 6, under the heading “Director of Laboratory,” estimates a regression in which the dependent variable equals one if the respondent added the director of the research lab in which they worked as an honorary author and equals zero if this was not the reason. The second model indicates those who added an author because he or she was in a position of authority and so forth. The estimated coefficients appear in Table 6 and the odds ratios are reported in S1 Appendix, Table E. Note the sample size is smaller for these regressions because we include only those respondents who say they have added a superfluous author to a manuscript.

**Table 6:** Reasons authors added to manuscripts: Estimated coefficients.

	Director of Laboratory		Position of Authority		Mentor	
	Coef.	Std. err	Coef.	Std. err	Coef.	Std. err
***Academic Ranks***						
**Assistant Professor**	-0.004	0.116	0.464**	0.128	0.925**	0.161
**Associate Professor**	0.047	0.110	0.383**	0.126	0.208	0.176
**Lecturer**	-0.369	0.331	0.374	0.298	1.373**	0.291
**Research Faculty**	0.414	0.211	0.487*	0.251	0.539	0.324
**Clinical Faculty**	0.352	0.309	-0.373	0.372	1.148**	0.347
**Other Rank**	0.325	0.238	0.249	0.268	0.488	0.304
***Gender and number of coauthors***						
**Male**	0.084	0.094	-0.075	0.106	-0.0207	0.134
**Number of Co-authors**	0.004	0.015	-0.035	0.021	-0.214**	0.036
***Disciplines***						
**Medicine**	0.863**	0.125	0.517**	0.116	0.374*	0.167
**Nursing**	0.371*	0.187	0.748**	0.162	0.846**	0.200
**Accounting**	-0.907	0.698	0.378	0.419	-0.578	0.703
**Economics**	-0.469	0.259	0.067	0.212	0.441	0.238
**Finance**	-1.635	0.965	0.219	0.441	-0.165	0.593
**Info systems**	0.110	0.435	0.327	0.389	1.032**	0.391
**Management**	-0.344	0.218	0.508**	0.162	0.691**	0.190
**Marketing**	-0.267	0.350	-0.053	0.309	0.541*	0.323
**Political Science**	-0.877	0.500	0.256	0.318	-0.721	0.573
**Psychology**	0.850**	0.154	-0.327	0.182	0.338	0.207
**Sociology**	0.100	0.233	-0.227	0.241	-0.511	0.345
**Biology**	0.961**	0.142	-0.726**	0.193	-0.453	0.256
**Chemistry**	0.858**	0.213	-0.916**	0.354	-0.505	0.416
**Computer Science**	-0.919*	0.412	0.393	0.265	0.093	0.374
**Ecology**	0.694**	0.207	-0.104	0.253	0.320	0.313
**Engineering**	0.704**	0.172	-0.246	0.209	-0.374	0.304
**Mathematics**	-0.228	0.463	-0.483	0.506	-1.30	0.965
**Physics**	0.100	0.242	-0.333	0.290	-0.074	0.389
***Publication history***						
**Publications**	0.005	0.003	-0.006	0.004	-0.005	0.006
**Constant**	-1.883**	0.137	-1.663**	0.142	-1.955**	0.203
	n = 3158; χ^2^ = 138.1		n = 3158; χ^2^ = 136.4		n = 3158; χ^2^ = 192.75	

Logit regression, dependent variable is binary: 1 = added director of laboratory as co-author, or someone in position of authority, or a mentor (even though they were not materially involved in the research), 0 = some other reason for adding author. Independent variables include academic ranks, disciplines, gender, number of co-authors, and number of publications in last five years.

* Indicates significance at the 5% level;

** significant at the 1% level.

The results are as expected. The individuals who are more likely to add a director of a laboratory are research faculty (they mostly work in research labs and centers), and scholars in fields in which laboratory work is a primary method of conducting research (medicine, nursing, psychology, biology, chemistry, ecology, and engineering). The second model suggests that the scholars who add an author because they feel pressure from individuals in a position of authority are junior faculty (assistant and associate professors, and research faculty) and individuals in medicine, nursing, and management. The third model suggests assistant professors, lecturers, research faculty, and clinical faculty are more likely to add their mentors as an honorary author. Since many mentorships are established in graduate school or through post-docs, it is sensible that scholars who are early in their career still feel an obligation to their mentors and are more likely to add them to manuscripts. Finally, the disciplines most likely to add mentors to manuscripts seem to be the “professional” disciplines: medicine, nursing, and business (economics, information systems, management, and marketing). We do not report the results for the other five reasons for adding honorary authors because few respondent characteristics were statistically significant. One explanation for this lack of significance may be the smaller sample size (less than 10% of the respondents indicated one of these remaining reasons as being the primary reason they added an author) or it may be that even if these rationales are relatively common, they might be distributed randomly across ranks and disciplines.

Turning to grant proposals, the dominant reason for adding authors to grant proposals even though they are not actually involved in the research was reputation. Of the more than 2100 individuals who gave a specific answer to this question, 60.8% selected “this individual had a reputation that increases the chances of the work being funded.” The second most frequently reported reason for grants was that the added individual was the director of the lab (13.5%), and third was people holding a position of authority (13%). All other reasons garnered a small number of responses.

We estimate a set of regressions similar to Table 6 using the reasons for honorary grant proposal authorship as the dependent variable and the independent variables from the grant proposal models of Table 5. Before estimating those models we also add six dummy variables reflecting different sources of research funding to see if the reason for adding honorary citations differs by type of funding. These dummy variables indicate funding from NSF, HHS (which includes the NIH), research grants from private corporations, grants from private, non-profit organizations, state research grants, and then a variable capturing all other federally funded grants. The omitted category is all other grants. The estimated coefficients appear in Table 7 and the odds ratios are reported in Table F in S1 Appendix.

**Table 7:** Reasons authors are added to grant proposals: Estimated coefficients.

Variables	Added author		Reputation		Director		Authority	
	Coef.	Std. err	Coef.	Std. err	Coef.	Std. err	Coef.	Std. err
***Academic Ranks and Gender***								
**Assistant**	0.54**	0.09	0.31*	0.14	-0.45*	0.21	0.20	0.22
**Associate**	0.46**	0.08	0.22	0.13	-0.27	0.19	0.26	0.20
**Lecturer**	0.97**	0.23	0.36	0.37	-1.23	0.76	0.24	0.57
**Res. faculty**	0.87**	0.16	-0.15	0.24	0.68*	0.28	-0.22	0.43
**Clinic faculty**	-0.12	0.38	-0.60	0.53	0.09	0.67	1.02	0.68
**Other rank**	0.91**	0.19	0.24	0.31	-0.08	0.43	0.46	0.43
**Male**	-0.30**	0.07	0.07	0.11	-0.06	0.16	-0.12	0.17
***Disciplines***								
**Medicine**	1.37**	0.09	0.28	0.15	0.04	0.21	-0.06	0.23
**Accounting**	0.40	0.28	0.01	0.50	-1.03	0.98	-0.71	0.99
**Economics**	0.34**	0.13	0.18	0.23	-0.03	0.34	-0.67	0.43
**Finance**	0.25	0.34	-0.48	0.62	0.45	0.77	0.69	0.77
**Info systems**	-0.11	0.34	0.04	0.61	0.36	0.76	1.01	0.67
**Management**	0.53**	0.17	0.73**	0.24	-0.70	0.41	-0.15	0.37
**Marketing**	0.09	0.29	-0.16	0.52	1.72**	0.54	-0.79	0.99
**Poly science**	-0.56**	0.20	0.59	0.40	-0.15	0.60	0.13	0.53
**Psychology**	-0.07	0.10	0.06	0.19	0.34	0.26	-0.52	0.34
**Sociology**	-0.06	0.14	0.02	0.26	-0.58	0.46	-0.44	0.46
**Biology**	-0.70**	0.10	-0.23	0.19	0.01	0.29	0.11	0.28
**Chemistry**	-0.53**	0.14	-0.31	0.26	0.70*	0.32	0.06	0.41
**Comp Science**	-0.01	0.17	0.05	0.31	-0.84	0.59	0.16	0.42
**Ecology**	-0.24	0.16	-0.30	0.29	0.04	0.40	0.14	0.40
**Engineer**	0.17	0.11	0.02	0.19	0.03	0.28	0.21	0.27
**Mathematics**	-0.53*	0.25	-0.30	0.46	omitted		0.64	0.56
**Physics**	-0.34*	0.16	-0.13	0.29	-0.27	0.44	0.31	0.39
***Grant history***								
**Number Grants**	0.03**	0.01	0.01	0.01	-0.02	0.01	-0.01	0.01
**Grant dollars**	-.15E-9	.23E-9	-4.4E-9	4.7E-9	1.7E-9	1.E-9	-4E-9	1.4E-8
**NSF**	0.54**	0.11	-0.09	0.21	0.49	0.33	0.11	0.30
**HHS**	1.15**	0.12	0.63**	0.21	-0.09	0.34	-0.60	0.33
**Corporation $**	0.45*	0.21	-0.92**	0.34	1.24**	0.44	0.39	0.45
**Nonprofit**	0.03	0.13	-0.09	0.24	0.62	0.38	-0.23	0.38
**State funding**	0.26	0.15	-0.03	0.27	0.58	0.40	-0.64	0.47
**Otr. FED Grants**	0.63**	0.14	-0.20	0.26	0.41	0.39	0.33	0.36
**Constant**	-2.11**	0.12	-0.20	0.21	-1.8**	0.35	-1.94	0.33
	n = 6343; χ^2^ = 893.4		n = 1711; χ^2^ = 109.0		n = 1693; χ^2^ = 70.6		n = 1711;χ^2^ = 44.6	

Logit regression, dependent variable is binary: 1 = added an author to a grant proposal because of his or reputation, or added the director of laboratory as co-author, or someone in position of authority (even though they were not materially involved in the research), 0 = some other reason for adding author. Independent variables include academic ranks, disciplines, gender, funding agency, number of grants, and total grand funding received in last five years.

* Indicates significance at the 5% level;

** significant at the 1% level.

The first column of results in Table 7 replicates and adds to the model in Table 5, in which the dependent variable is: “have you added honorary authors to grant proposals.” The reason we replicate that model is to add the six funding sources to the regression to see if some agencies see more honorary authors in their proposals than other agencies. The results in Table 7 suggest they do. Federally funded grants are more likely to have honorary authorships than other sources of grant funding as the coefficients on NSF, NIH, and other federal funding are all positive and significant at the 0.01 level. Corporate research grants also tend to have honorary authors included.

The remaining columns in Table 7 suggest that scholars in medicine and management are more likely to add honorary authors to grant proposals because of the added scholar’s reputation, but there is little statistical difference across the other characteristics of our respondents. Exploring the different sources of funds, adding an individual because of his or her reputation is more likely to be practiced with grants to the Department of Health and Human Services (probably because of the heavy presence of medical proposals and honorary authorship is common in medicine) and it is statistically less likely to be used in grant proposals directed towards corporate research funding.

Table 7 shows that lab directors tend to be honorary authors in grant proposals with assistant professors and for grant proposals directed to private corporations. While position of authority (i.e., political power) was the third most frequently cited reason to add someone to a proposal, its practice seems to be dispersed across the academic universe as the regression results in Table 7 do not show much variation across rank, discipline, their past experience with research funding, or the funding source to which the proposal was directed. The remaining reasons for adding authors garnered a small portion of the total responses and there was little significant variation across the characteristics measured here. For these reasons, their regression results are not reported.

### Coercive citations

**Fig 3:**
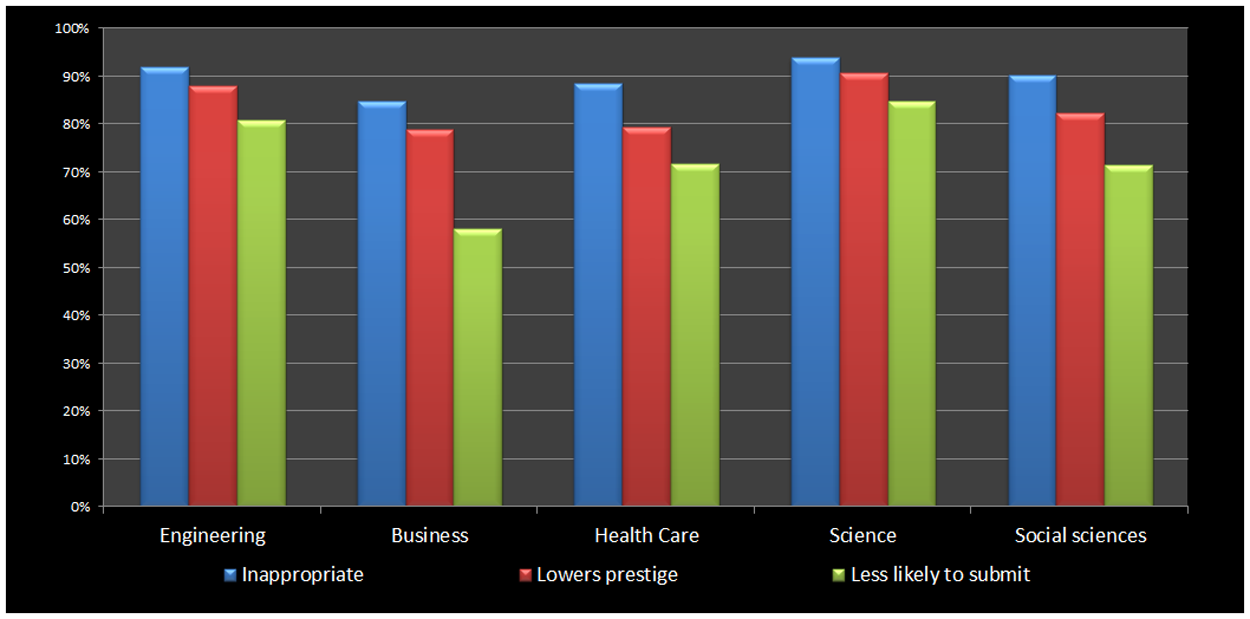
Disapproval of coercive citation by major academic group. The first column in each cluster presents the percentage of respondents from each major academic group who either strongly agree or agree with the statement the coercive citations, “is inappropriate.” The second column is the percentage that agrees to, “[it] reduces the prestige of the journal.” The third column reflects agreement to, “are less likely to submit work to a journal that coerces.”

There is widespread distaste among academics concerning the use of coercive citation. Over 90% of our respondents view coercion as inappropriate, 85.3% think its practice reduces the prestige of the journal, and 73.9% are less likely to submit work to a journal that coerces. These opinions are shared across the academic spectrum as shown in Fig 3, which breaks out these responses by the major fields, medicine, science, engineering, business, and the social sciences. Despite this disapproval, 14.1% of the overall respondents report being coerced. Similar to the analyses above, our task is to see if there is a systematic set of attributes of scholars who are coerced or if there are attributes of journals that are related to coercion.

Two dependent variables are used to measure the existence and the frequency of coercive citation. The first is a binary dependent variable, whether respondents were coerced or not, and the second counts the frequency of coercion, asking our respondents *how many times* they have been coerced in the last five years. Table 8 presents estimates of the logit model (coerced or not) and their odds ratios and Table 9 presents estimates of the negative binomial model (measuring the frequency of coercion) and their accompanying incident rate ratios. With but a single exception (the estimated coefficient on female scholars was opposite our expectation) our hypotheses are supported. In this sample, it is males who are more likely to be coerced, the effect size estimates that being a male raises the odds ratio of being coerced by 18%. In the frequency estimates in Table 9, however, there was no statistical difference between male and female scholars.

**Table 8:** Existence of coercive citation: Estimated coefficients and odds ratios.

Variables	Estimated coefficient	Std. error	Odds ratio	Std. error
***Academic Ranks***				
**Assistant professor**	0.357**	0.076	1.429**	0.109
**Associate professor**	0.195**	0.073	1.215**	0.089
**Lecturer**	-0.538*	0.212	0.584*	0.124
**Other faculty**	0.051	0.117	1.052	0.123
***Gender and number of coauthors***				
**Male**	0.164*	0.068	1.178*	0.080
**Number coauthors**	-0.096**	0.018	0.908**	0.016
***Disciplines***				
**Medicine**	-0.493**	0.089	0.610**	0.055
**Nursing**	-0.524**	0.153	0.592**	0.090
**Accounting**	0.535**	0.157	1.708**	0.268
**Economics**	0.235*	0.102	1.265*	0.129
**Finance**	1.281**	0.131	3.601**	0.472
**Info systems**	1.306**	0.099	3.691**	0.364
**Management**	1.166**	0.088	3.208**	0.281
**Marketing**	1.364**	0.093	3.911**	0.362
**Political science**	-0.942**	0.235	0.390**	0.091
**Psychology**	-0.621**	0.116	0.537**	0.062
**Sociology**	-0.377*	0.138	0.686**	0.094
**Biology**	-0.114	0.198	0.892	0.177
**Chemistry**	-0.886**	0.154	0.412**	0.063
**Computer science**	-0.448*	0.173	0.639**	0.111
**Ecology**	0.778**	0.158	2.178**	0.344
**Engineering**	0.582**	0.090	1.789**	0.160
**Mathematics**	-1.625**	0.321	0.197**	0.063
**Physics**	-1.215**	0.225	0.297**	0.067
***Publication history***				
**Total publications**	0.028**	0.002	1.028**	0.002
**Constant**	-2.190**	0.099	0.112	0.011**
	n = 11567; χ^2^ = 1022.9			

Logit regression, dependent variable is binary: 1 = have been coerced to add citations, 0 = have not been coerced. Independent variables include academic ranks, disciplines, gender, number of co-authors, and number of publications in last five years.

* Indicates significance at the 5% level;

** significant at the 1% level.

**Table 9:** Frequency of coercive citation: Estimated coefficients and incidence rate ratios.

Variables	Estimated Coefficient	Std. error	IncidenceRate Ratio	Std. error
***Academic Ranks***				
**Assistant professor**	0.281**	0.074	1.324**	0.097
**Associate professor**	0.094	0.070	1.099	0.077
**Lecturer**	-0.148	0.179	0.862	0.155
**Other faculty**	0.055	0.114	1.056	0.120
***Gender and number of coauthors***				
**Male**	0.067	0.064	1.070	0.069
**Number coauthors**	-0.037*	0.018	0.964*	0.017
***Disciplines***				
**Medicine**	1.946**	0.135	7.004**	0.948
**Nursing**	1.839**	0.252	6.287**	1.584
**Accounting**	0.233	0.149	1.262	0.188
**Economics**	0.051	0.094	1.052	0.099
**Finance**	0.987**	0.125	2.684**	0.336
**Info systems**	0.930**	0.094	2.535**	0.239
**Management**	0.882**	0.083	2.415**	0.200
**Marketing**	1.015**	0.088	2.760**	0.242
**Political science**	-1.115**	0.205	0.328**	0.067
**Psychology**	-0.848**	0.101	0.428**	0.043
**Sociology**	-0.590**	0.123	0.554**	0.068
**Biology**	-0.477**	0.175	0.621**	0.109
**Chemistry**	-0.923**	0.121	0.397**	0.048
**Computer science**	-0.736**	0.152	0.479**	0.073
**Ecology**	0.211	0.151	1.235	0.187
**Engineering**	0.280**	0.082	1.323**	0.108
**Mathematics**	-2.010**	0.274	0.134**	0.037
**Physics**	-1.676**	0.200	0.187**	0.037
***Publication history***				
**Total publications**	0.032**	0.002	1.032**	0.002
**Constant**	-1.659**	0.098	0.190**	0.019
	n = 8951; χ^2^ = 1071.1			

Negative binomial regression, dependent variable is number of times respondents report being coerced for citations in last five years. Independent variables include academic ranks, disciplines, gender, number of co-authors, and number of publications in last five years.

* Indicates significance at the 5% level;

** significant at the 1% level.

Consistent with our hypotheses, assistant professors and associate professors were more likely to be coerced than full professors and the effect was larger for assistant professors. Being an assistant professor increases the odds that you will be coerced by 42% over a professor while associate professors see about half of that, a 21% increase in their odds. Table 9 shows assistant professors are also coerced more frequently than professors. Co-authors had a negative and significant coefficient as predicted in both sets of results. Consequently, comparing Tables 3 and 8 we see that manuscripts with many co-authors are more likely to add honorary authors, but are less likely to be targeted for coercion. Finally, we find significant variation across disciplines. Eight disciplines are significantly more likely to be coerced than the average across all disciplines and ordered by their odds ratios (largest to smallest) they are: marketing, information systems, finance, management, ecology, engineering, accounting, and economics. Nine disciplines are less likely to be coerced and ordered by their odds ratios (smallest to largest) they are: mathematics, physics, political science, chemistry, psychology, nursing, medicine, computer science, and sociology. Again, there is support for our speculation that disciplines in which grant funding is less critical (and therefore publication is relatively more critical) experience more coercion. In the top coercion category, six of the eight disciplines are business disciplines, where research funding is less common, and in “less than average” coercion disciplines, six of the nine disciplines rely heavily on grant funding. The anomaly (and one that deserves greater study) is that the social sciences see less than average coercion even though publication is their primary measure of academic success. While they are prime targets for coercion, the editors in their disciplines have largely resisted the temptation. Again, this same pattern emerges in the frequency model. In the S1 Appendix, these models are re-estimated after standardizing the continuous variables. Results appear in Table G (existence of coercion) and Table H (frequency of coercion.)

### Coercive citations: Journal data

To achieve a deeper understanding of coercive citation, we reexamine this behavior using academic journals as our unit of observation. We analyze these journal-based data in two ways: 1) a logit model in which the dependent variable equals 1 if that journal was named as having coerced and 0 if not and 2) a negative binomial model where the dependent variable is the count of the number of times a journal was identified as one where coercion occurred. As before, the variance of these data substantially exceeds the mean and thus Poison regression is inappropriate. To test our hypotheses, our included independent variables are the dummy variables for discipline, journal rank, and dummy variables for different types of publishers. We control for some of the different editorial practices across journals by including the number of documents published annually by each journal and the average number of citations per article.

The results of the journal-based analysis appear in Table 10. Once again, and consistent with our hypothesis, the differences across disciplines emerge and closely follow the previous results. The discipline journals most likely to have coerced authors for citations are in business. The effect of a journal’s rank on its use of coercion is perhaps the most startling finding. Measuring journal rank using the h-index suggests that more highly rated journals are more likely to have coerced and coerced more frequently, which is opposite our hypothesis that lower ranked journals are more likely to coerce. Perhaps the chance to move from being a “good” journal to a “very good” journal is just too tempting to pass. There is some anecdotal evidence that is consistent with this result. If one surfs through the websites of journals, many simply do not mention their rank or impact factor. However, those that do mention their rank or impact tend to be more highly ranked journals (a low-ranked journal typically doesn’t advertise that fact), but the very presence of the impact factor on a website suggests that the journal, or more importantly the publisher, places some value on it and, given that pressure, it is not surprising to find that it may influence editorial decisions. On the other hand, we might be observing the results of established behavior. If some journals have practiced coercion for an extended time then their citation count might be high enough to have inflated their h-index. We cannot discern a direction of causality, but either way our results suggest that more highly ranked journals end up using coercion more aggressively, all else equal.

**Table 10:** Journals that have coerced: Estimated coefficients, odds ratios, and incident rate ratios (all journals).

	Coerced Authors (logit)				Frequency Coerced Authors (negative binomial)			
	Coefs.	Std. Error	Odds ratios	Std. Error	Coefs.	Std. error	IRR	Std. Error
***Journal Attributes***								
TotDocs	0.0002**	0.000	1.000**	0.0001	0.0002	0.0001	1.000	0.0001
RefperDoc	0.001	0.001	1.001	0.001	0.004	0.002	1.004	0.002
University	1.909	1.074	6.750	7.252	1.597	1.120	4.940	5.533
Academic	2.386*	1.062	10.867*	11.548	2.183*	1.108	8.870*	9.830
Private	2.625*	1.060	13.812*	14.639	2.421*	1.103	11.254*	12.419
***Disciplines***								
Medicine	-1.633**	0.136	0.195**	0.026	-1.862**	0.143	0.155**	0.022
Nursing	0.271	0.281	1.311	0.369	-0.070	0.335	0.932	0.313
Accounting	1.097	0.587	2.994	1.759	1.656**	0.608	5.240**	3.188
Economics	0.570**	0.204	1.768**	0.362	0.922**	0.210	2.515**	0.529
Finance	1.693**	0.205	5.437**	1.113	1.662**	0.266	5.272**	1.402
Info Sys	1.669**	0.259	5.307**	1.376	1.592**	0.342	4.914**	1.680
Management	1.111**	0.148	3.037**	0.449	0.966**	0.180	2.628**	0.473
Marketing	2.110**	0.382	8.251**	3.156	2.871**	0.508	17.651**	8.977
Polysci	-1.056	0.561	0.348	0.195	-0.528	0.415	0.590	0.245
Psychology	-0.040	0.185	0.961	0.178	-0.077	0.198	0.925	0.183
Sociology	-0.836**	0.179	0.433**	0.078	-0.950**	0.182	0.387**	0.070
Biology	-1.850**	0.176	0.157**	0.028	-2.209**	0.191	0.110**	0.021
Chemistry	-0.913**	0.222	0.401**	0.089	-1.191**	0.254	0.304**	0.077
CompSci	-0.321	0.196	0.725	0.142	0.140	0.188	1.151	0.216
Ecology	-0.476**	0.180	0.621**	0.112	-0.731**	0.192	0.481**	0.092
Engineering	-0.416**	0.149	0.659**	0.098	-0.384*	0.152	0.681*	0.104
Mathematics	-0.400	0.213	0.670	0.143	-0.670**	0.231	0.511**	0.118
Physics	-0.580**	0.198	0.560**	0.111	-1.138**	0.248	0.320**	0.080
***Journal Rankings***								
h-Index	0.022**	0.001	1.022**	0.001	0.031**	0.002	1.000**	0.0002
Constant	-6.128**	1.063	0.002**	0.002	-6.033**	1.106	1.004**	0.002
	N = 16,651; χ^2^ = 873.42				N = 16,651; χ^2^ = 778.55			

The unit of observation is a journal. The dependent variable for the logit model is binary: 1 = journal named as having coerced, 0 = not so named, and for the frequency model the dependent variable is the number of times a journal was named as one that has coerced. Independent variables include the total number of documents published by the journal in a year, the average references per document, the type of publisher, academic disciplines, and the journal’s ranking as measured by the h-index.

* Indicates significance at the 5% level;

** significant at the 1% level.

There seems to be publisher effects as well. As predicted, journals published by private, profit oriented companies are more likely to be journals that have coerced, but it also seems to be more common in the academic associations than university publishers. Finally, we note that the total number of documents published per year is positively related to a journal having coerced and the impact of the average number of citations per document was not significantly different than zero.

The result that higher-ranked journals seem to be more inclined than lower-ranked journals to have practiced coercion warrants caution. These data contain many obscure journals; for example, there are more than 4000 publications categorized as medical journals and this long tail could create a misleading result. For instance, suppose some medical journals ranked between 1000–1200 most aggressively use the practice of coercion. In relative terms these are “high” ranked journals because 65% of the journals are ranked even lower than these clearly obscure publications. To account for this possibility, a second set of estimates was calculated after eliminating all but the “top-30” journals in each discipline. The results appear in Table 11 and generally mirror the results in Table 10. Journals in the business disciplines are more likely to have used coercion and used it more frequently than the other disciplines. Medicine, biology, and computer science journals used coercion less. However, even concentrating on the top 30 journals in each field, the h-index remains positive and significant; higher ranked journals in those disciplines are more likely to have coerced.

**Table 11:** Journals that have coerced, top 30 journals: Estimated coefficients, odds ratios, and incident rate ratios.

	Coerced Authors (logit)				Frequency Coerced Authors (negative binomial)			
	Coefs.	Std. Error	Odds Ratio	Std. Error	Coefs.	Std. Error	IRR	Std. Error
***Journal Attributes***								
**TotDocs**	0.001	0.001	1.000	0.001	0.001	0.001	1.000	0.001
**RefperDoc**	-0.008*	0.004	0.992*	0.004	-0.009	0.005	0.991	0.005
**University**	-0.049	0.433	0.952	0.412	-0.471	0.458	0.624	0.286
**Academic**	0.297	0.250	1.346	0.337	0.248	0.248	1.282	0.317
***Disciplines***								
**Medicine**	-2.780**	0.757	0.062**	0.047	-2.461**	0.662	0.085**	0.056
**Nursing**	0.565	0.460	1.760	0.810	0.052	0.499	1.054	0.526
**Accounting**	0.073	0.650	1.075	0.699	0.678	0.533	1.969	1.050
**Economics**	-0.295	0.543	0.744	0.404	-0.794	0.598	0.452	0.270
**Finance**	1.998**	0.400	7.373**	2.953	1.991**	0.370	7.324**	2.709
**Info systems**	1.108**	0.423	3.027**	1.282	1.675**	0.371	5.339**	1.984
**Management**	2.207**	0.406	9.093**	3.692	2.019**	0.362	7.531**	2.723
**Marketing**	1.504**	0.439	4.502**	1.975	2.823**	0.353	16.836**	5.944
**Polysci**	-0.556	0.734	0.574	0.421	-0.068	0.573	0.934	0.536
**Psychology**	0.037	0.441	1.038	0.457	-0.441	0.479	0.643	0.308
**Sociology**	0.212	0.463	1.236	0.572	0.120	0.444	1.128	0.501
**Biology**	-2.108**	0.687	0.121**	0.083	-1.974**	0.669	0.139**	0.093
**Chemistry**	-0.445	0.458	0.641	0.293	-1.046*	0.479	0.351*	0.168
**CompSci**	-2.577**	0.979	0.076**	0.074	-3.009**	1.011	0.049**	0.050
**Ecology**	0.129	0.404	1.137	0.459	1.027**	0.360	2.794**	1.007
**Engineering**	0.273	0.395	1.314	0.520	0.051	0.384	1.052	0.404
**Mathematics**	0.315	0.427	1.371	0.585	-0.203	0.446	0.816	0.364
**Physics**	0.339	0.438	1.403	0.615	-0.442	0.458	0.643	0.294
***Journal Ranking***								
**h-Index**	0.011**	0.003	1.011**	0.003	0.012**	0.003	1.012**	0.003
**Constant**	-2.073**	0.351	0.126	0.044	-1.705	0.388	0.182	0.071
	N = 540; χ^2^ = 73.87				N = 540; χ^2^ = 127.21			

Table 11 repeats analysis in Table 10 cutting sample to include only the top 30 journals in each discipline as measured by the h-index. The dependent variable for the logit model is binary: 1 = journal named as having coerced, 0 = not so named, and for the frequency model the dependent variable is the number of times a journal was named as one that has coerced. Independent variables include the total number of documents published by the journal in a year, the average references per document, the type of publication, academic disciplines, and the journal’s ranking as measured by the h-index.

* Indicates significant at the 5% level;

** significant at the 1% level.

### Padded reference lists

Our final empirical tests focus on padded citations. We asked our respondents that if they were submitting an article to a journal with a reputation of asking for citations even if those citations are not critical to the content of the article, would you “add such citations BEFORE SUBMISSION.” Again, more than 40% of the respondents said they agreed with that sentiment. Regarding grant proposals, 15% admitted to adding citations to their reference list in grant proposals “even if those citations are of marginal import to my proposal.”

To see if reference padding is as systematic as the other types of manipulation studied here, we use the categorical responses to the above questions as dependent variables and estimate ordered logit models using the same descriptive independent variables as before. The results for padding references in manuscripts and grant proposals appear in Tables 12 and 13, respectively. Once more, with minor deviation, our hypotheses are strongly supported.

**Table 12:** Padding citations in manuscripts: Estimated coefficients and odds ratios.

Variables	Estimated coefficients	Std. error	Odds ratios	Std. error
***Academic Ranks***				
**Assistant Professor**	0.752**	0.047	2.121**	0.100
**Associate Professor**	0.479**	0.045	1.615**	0.073
**Lecturer**	0.593**	0.109	1.810**	0.196
**Other faculty**	0.420**	0.062	1.522**	0.094
***Gender and number of co-authors***				
**Male**	-0.325**	0.040	0.722**	0.028
**Coauthors**	0.008	0.008	1.007	0.008
***Disciplines***				
**Medicine**	-0.913**	0.044	0.401**	0.018
**Nursing**	-1.168**	0.071	0.311**	0.022
**Accounting**	0.968**	0.113	2.632**	0.298
**Economics**	0.628**	0.066	1.874**	0.123
**Finance**	0.551**	0.111	1.734**	0.192
**Information Systems**	0.539**	0.081	1.714**	0.139
**Management**	0.862**	0.071	2.367**	0.167
**Marketing**	0.913**	0.078	2.493**	0.195
**Political Science**	0.440**	0.101	1.553**	0.156
**Psychology**	0.280**	0.058	1.323**	0.076
**Sociology**	0.562**	0.072	1.754**	0.126
**Biology**	-0.704**	0.105	0.495**	0.052
**Chemistry**	-0.452**	0.065	0.636**	0.042
**CompSci**	-0.450**	0.090	0.637**	0.057
**Ecology**	-0.169	0.105	0.844	0.089
**Engineering**	-0.384**	0.062	0.618**	0.042
**Mathematics**	-0.752**	0.094	0.471**	0.044
**Physics**	-0.750**	0.082	0.472**	0.039
***Publication history***				
**Publications**	-0.003*	0.001	0.997*	0.001
**Aware of coercion**	0.398**	0.042	1.489**	0.062
**Coerced**	0.451**	0.058	1.570**	0.091
n = 11,518; *χ*^2^ = 2262.9				

Ordered logit regression, dependent variable is categorical: 1 = strongly disagree, 2 = disagree, 3 = neutral, 4 = agree, 5 = strongly agree; to the likelihood of padding citations (see survey in supplemental materials). Independent variables include academic ranks, disciplines, gender, number of co-authors, number of publications, and awareness of editorial coercion.

* Indicates significance at the 5% level;

** significant at the 1% level.

**Table 13:** Padding citations in grant proposals: Estimated coefficients and odds ratios.

Variables	Estimated coefficients	Std. error	Odds ratios	Std. errors
***Academic Ranks***				
**Assistant Professor**	0.346**	0.059	1.413**	0.084
**Associate Professor**	0.072	0.053	1.075	0.057
**Lecturer**	0.615**	0.169	1.849**	0.313
**Other faculty**	-0.154	0.148	0.857	0.127
**Research fac.**	0.209	0.126	1.232	0.155
**Clinical fac.**	0.584**	0.196	1.793**	0.352
***Gender***				
**Male**	0.213**	0.049	1.238**	0.061
***Disciplines***				
**Medicine**	0.050	0.054	1.051	0.057
**Nursing**	-0.272**	0.087	0.762**	0.066
**Accounting**	0.437*	0.204	1.547*	0.315
**Economics**	0.536**	0.092	1.710**	0.158
**Finance**	1.277**	0.235	3.588**	0.844
**Information Systems**	-0.114	0.249	0.892	0.222
**Management**	0.752**	0.102	2.121**	0.216
**Marketing**	0.668**	0.200	1.951**	0.390
**Political Science**	0.182	0.116	1.199	0.139
**Psychology**	-0.496**	0.074	0.609**	0.045
**Sociology**	-0.244*	0.101	0.784*	0.079
**Biology**	-0.190**	0.065	0.827**	0.053
**Chemistry**	-0.183*	0.090	0.833*	0.075
**CompSci**	-0.421**	0.129	0.656**	0.085
**Ecology**	-0.270**	0.106	0.763**	0.080
**Engineering**	-0.434**	0.085	0.648**	0.055
**Mathematics**	-0.728**	0.153	0.483**	0.074
**Physics**	-0.550**	0.102	0.577**	0.059
***Grant history***				
**# grants**	-0.002	0.003	0.998	0.003
**Grant dollars**	0.000	0.000	1.000	1.1E-09
**Added authors**	0.798**	0.052	2.221**	0.116
	n = 7487; *χ*^2^ = 620.6			

Ordered logit regression, dependent variable is categorical: 1 = strongly disagree, 2 = disagree, 3 = neutral, 4 = agree, 5 = strongly agree; to the likelihood of padding citations (see survey in supplemental materials). Independent variables include academic ranks, disciplines, gender, number of co-authors, number of grants received, total grant money received in last 5 years, and awareness of editorial coercion.

* Indicates significance at the 5% level;

** significant at the 1% level.

Tables 12 and 13 show that scholars of lesser rank and those without tenure are more likely to pad citations to manuscripts and skew citations in grant proposals than are full professors. The gender results are mixed, males are less likely to pad their citations in manuscripts, but more likely to pad references in grant proposals. It is the business disciplines and the social sciences that are more likely to pad their references in manuscripts and business and medicine who pad citations on grant proposals. In both situations, familiarity with other types of manipulation has a strong, positive correlation with the likelihood that individuals pad their reference list. That is, respondents who are aware of coercive citation and those who have been coerced in the past are much more likely to pad citations before submitting a manuscript to a journal. And, scholars who have added honorary authors to grant proposals are also more likely to skew their citations to high-impact journals. While we cannot intuit the direction of causation, we show evidence that those who manipulate in one dimension are willing to manipulate in another.

## Discussion

Our results are clear; academic misconduct, specifically misattribution, spans the academic universe. While there are different levels of abuse across disciplines, we found evidence of honorary authorship, coercive citation, and padded citation in every discipline we sampled. We also suggest that a useful construct to approach misattribution is to assume individual scholars make deliberate decisions to cheat after weighing the costs and benefits of that action. We cannot claim that our construct is universally true because other explanations may be possible, nor do we claim it explains all misattribution behavior because other factors can play a role. However, the systematic pattern of superfluous authors, coerced citations, and padded references documented here is consistent with scholars who making deliberate decisions to cheat after evaluating the costs and benefits of their behavior.

Consider the use of honorary authorship in grant proposals. Out of the more than 2100 individuals who gave a specific reason as to why they added a superfluous author to a grant proposal, one rationale outweighed the others; over 60% said they added the individual because of they thought the added scholar’s reputation increased their changes of a positive review. That behavior, adding someone with a reputation even though that individual isn’t expected to contribute to the work was reported across disciplines, academic ranks, and individuals’ experience in grant work. Apparently, adding authors with highly recognized names to grant proposals has become part of the game and is practiced across disciplines and rank.

Focusing on manuscripts, there is more variation in the stated reasons for honorary authorship. Lab directors are added to papers in disciplines that are heavy lab users and junior faculty members are more likely to add individuals in positions of authority or mentors. Unlike grant proposals, few scholars add authors to manuscripts because of their reputation. A potential explanation for this difference is that many grant proposals are not blind reviewed, so grant reviewers know the research team and can be influenced by its members. Journals, however, often have blind referees, so while the reputation of a particular author might influence an editor it should not influence referees. Furthermore, this might reflect the different review process of journals versus funding agencies. Funding agencies specifically consider the likelihood that a research team can complete a project and the project’s probability of making a significant contribution. Reputation can play a role in setting that perception. Such considerations are less prevalent in manuscript review because a submitted work is complete—the refereeing question is whether it is done well and whether it makes a significant contribution.

Turning to coercive citations, our results in Tables 8 and 9 are also consistent with a model of coercion that assumes editors who engage in coercive citation do so mindfully; they are influenced by what others in their field are doing and if they coerce they take care to minimize the potential cost that their actions might trigger. Parallel analyses using a journal data base are also consistent with that view. In addition, the distinctive characteristics of each dataset illuminate different parts of the story. The author-based data suggests editors target their requests to minimize the potential cost of their activity by coercing less powerful authors and targeting manuscripts with fewer authors. However, contrary to the honorary authorship results, females are less likely to be coerced than males, *ceteris paribus*. The journal-based data adds that it is higher-ranked journals that seem to be more inclined to take the risk than lower ranked journals and that the type of publisher matters as well. Furthermore, both approaches suggest that certain fields, largely located in the business professions, are more likely to engage in coercive activities. This study did not investigate why business might be more actively engaged in academic misconduct because there was little theoretical reason to hypothesize this relationship. There is however some literature suggesting that ethics education in business schools has declined [34]. For the last 20–30 years business schools have turned to the mantra that stock holder value is the only pertinent concern of the firm. It is a small step to imagine that citation counts could be viewed as the only thing that matters for journals, but additional research is needed to flesh out such a claim.

Again, we cannot claim that our cost-benefit model of editors who try to inflate their journal impact factor score is the only possible explanation of coercion. Even if editors are following such a strategy, that does not rule out additional considerations that might also influence their behavior. Hopefully future research will help us understand the more complex motivations behind the decision to manipulate and the subsequent behavior of scholars.

Finally, it is clear that academics see value in padding citations as it is a relatively common behavior for both manuscripts and grants. Our results in Tables 12 and 13 also suggest that the use of honorary authorship and padding citations in grant proposals and coercive citation and padding citations in manuscripts is correlated. Scholars who have been coerced are more likely to pad citations before submitting their work and individuals who add authors to manuscripts also skew their references on their grant proposals. It seems that once scholars are willing to misrepresent authorship and/or citations, their misconduct is not limited to a single form of misattribution.

It is difficult to examine these data without concluding that there is a significant level of deception in authorship and citation in academic research and while it would be naïve to suppose that academics are above such scheming to enhance their position, the results suggest otherwise. The overwhelming consensus is that such behavior is inappropriate, but its practice is common. It seems that academics are trapped; compelled to participate in activities they find distasteful. We suggest that the fuel that drives this cultural norm is the competition for research funding and high-quality journal space coupled with the intense focus on a single measure of performance, the number of publications or grants. That competition cuts both ways, on the one hand it focuses creativity, hones research contributions, and distinguishes between significant contributions and incremental advances. On the other hand, such competition creates incentives to take shortcuts to inflate ones’ research metrics by strategically manipulating attribution. This puts academics at odds with their core ethical beliefs.

The competition for research resources is getting tighter and if there is an advantage to be gained by misbehaving then the odds that academics will misbehave increase; left unchecked, the manipulation of authorship and citation will continue to grow. Different types of attribution manipulation continue to emerge; citation cartels (where editors at multiple journals agree to pad the other journals’ impact factor) and journals that publish anything for a fee while falsely claiming peer-review are two examples [30, 35].

It will be difficult to eliminate such activities, but some steps can probably help. Policy actions aimed at attribution manipulation need to reduce the benefits of manipulation and/or increase the cost. One of the driving incentives of honorary authorship is that the number of publications has become a focal point of evaluation and that number is not sufficiently discounted by the number of authors [36]. So, if a publication with x authors counted as 1/x publications for each of the authors, the ability to inflate one’s vita is reduced. There are problems of course, such as who would implement such a policy, but some of these problems can be addressed. For example if the online, automated citation counts (e.g., h-index, impact factor, calculators such as SCOPUS and Google Scholar) automatically discounted their statistics by the number of authors, it could eventually influence the entire academe. Other shortcomings of this policy is that this simple discounting does not allow for differential credit to be given that may be warranted, nor does it remove the power disparity in academic ranks. However, it does stiffen the resistance to adding authors and that is a crucial step.

An increasing number of journals, especially in medicine, are adopting authorship guidelines developed by independent groups, the most common being set forth by the International Committee of Medical Journal Editors (ICMJE) [37]. To date, however, there is little evidence that those standards have significantly altered behavior; although it is not clear if that is because authors are manipulating in spite of the rules, if the rules are poorly enforced, or if they are poorly designed from an implementation perspective [21]. Some journals require authors to specifically enumerate each author’s contribution and require all of the authors to sign off on that division of labor. Such delineation would be even more effective if authorship credit was weighted by that division of labor. Additional research is warranted.

There may be greater opportunities to reduce the practice of coercive citation. A fundamental difference between coercion and honorary authorship is the paper trail. Editors write down such “requests” to authors, therefore violations are easier to document and enforcement is more straightforward. First, it is clear that impact factors should no longer include self-citations. This simple act removes the incentive to coerce authors. Reuters makes such calculations and publishes impact factors including and excluding self-citations. However, the existence of multiple impact factors gives journals the opportunity to adopt and advertise the factor that puts them in the best light, which means that journals with editors who practice coercion can continue to use impact factors that can be manipulated. Thus, self-citations should be removed from all impact factor calculations. This does not eliminate other forms of impact factor manipulation such as posting accepted articles on the web and accumulating citations prior to official publication, but it removes the benefit of editorial coercion and other strategies based on inflating self-citation [38]. Second, journals should explicitly ban their editors from coercing. Some journals are taking these steps and while words do not insure practice, a code of ethics reinforces appropriate behavior because it more closely ties a journal’s reputation to the practices of its editors and should increase the oversight of editorial boards. Some progress is being made on the adoption of editorial guidelines, but whether they have any impact is currently unknown [39, 40].

These results also reinforce the idea that grant proposals be double blind-reviewed. Blind-review shifts the decision calculus towards the merit of a proposal and reduces honorary authorship incentives. The current system can inadvertently encourage misattribution. For example, scholars are often encouraged to visit granting agencies to meet with reviewers and directors of programs to talk about high-interest research areas. Such visits make sense, but it is easy for those scholars to interpret their visit as a name-collecting exercise; finding people to add to proposals and collecting references to cite. Fourth, academic administrators, Provosts, Deans, and Chairs need to have clear rules concerning authorship. Far too many of our respondents said they added a name to their work because that individual could have an impact on their career. They also need to have guidelines that address the inclusion of mentors and lab directors to author lists. Proposals that include name-recognizable scholars for only a small proportion of the grant should be viewed with suspicion. This is a consideration in some grant opportunities, but that linkage can be strengthened. Finally, there is some evidence that mentoring can be effective, but there is a real question as to whether mentors are teaching compliance or how to cheat [41].

There are limitations in this study. Although surveys have shortcomings such as self-reporting bias, self-selection issues, etc., there are some issues for which surveys remain as the data collection method of choice. Manipulation is one of these issues. It would be difficult to determine if someone added honorary authors or padded citations prior to submission without asking that individual. Similarly, coercion is most directly addressed by asking authors if editors coerced them for citations. Other approaches, such as examining archival data, running experiments, or building simulations, will not work. Thus, despite its shortcomings, survey is the method of choice.

Our survey was sent via email and the overall response rate was 10.5%, which by traditional survey standards may be considered to be low. We have no data on how many surveys were filtered as spam or otherwise ended up in junk mail folders or how many addresses were obsolete. We recognize however that there is a rising hesitancy by individuals to click on an emailed link and that is what we were asking our recipients to do. For these reasons, we anticipated that our response rate may be low and compensated by increasing the number of surveys sent out. In the end, we have over 12,000 responses and found thousands of scholars who have participated in manipulation. In the S1 appendix, Table A presents response rates by discipline and while there is variation across disciplines, that variation does not correlate with any of the fundamental results, that is, there does not seem to be a discipline bias arising from differential response rates.

A major concern when conducting survey research is that the sample may not represent the population. To address this possible issue in our study, we perform various statistical analyses to determine if we encountered sampling bias. First, we compared two population demographics (sex and academic rank) to the demographics of our respondents (see Table B in S1 Appendix). The percentage of males and females in each discipline was very close to the reported sex of the respondents. There was greater variation in academic ranks with the rank of full professor being over-represented in our sample. One should keep this in mind when interpreting our findings. However, our hypotheses and results suggest that professors are the least likely to be coerced, use padded citations, and use honorary authorship, consequently our results may actually under-estimate the incidence of manipulation. Perhaps the greatest concern of potential bias innate in surveys comes from the intuition that individuals who are more intimately affected by a particular issue are more likely to respond. In the current study, it is plausible that scholars who have been coerced, or felt obligated to add authors to manuscripts, or have added investigators to grants proposals, are upset by that consequence and more likely to respond. However, if that motivation biased our responses it should show up in the response rates across disciplines, i.e., disciplines reporting a greater incidence of manipulation should have higher percentage of their population experiencing manipulation and thus higher response rates. The rank correlation coefficient between discipline response rates and the proportion of scholars reporting manipulation is *r*_*s*_ = -0.181, suggesting virtually no relationship between the two measures.

In the end, we cannot rule out the existence of bias but we find no evidence that suggests it affects our results. We are left with the conclusion that scholars manipulate attribution adding honorary authors to their manuscripts and false investigators to their grant proposals, and some editors coerce scholars to add citations that are not pertinent to their work. It is unlikely that this unethical behavior can be totally eliminated because academics are a competitive, intelligent, and creative group of individuals. However, most of our respondents say they want to play it straight and therefore, by reducing the incentives of misbehavior and raising the costs of inappropriate attribution, we can expect a substantial portion of the community to go along. With this inherent support and some changes to the way we measure scientific contributions, we may reduce attribution misbehavior in academia [42].

## Supporting information

S1 Appendix Statistical methods, surveys, and additional results. (DOCX)

S2 Appendix Honorary authors data. (XLS)

S3 Appendix Coercive citation data. (XLS)

S4 Appendix Journal data. (XLS)
