# Supplementary material for: Authorship and citation manipulation in academic research
Source: PLoS One. 2017 Dec 6;12(12):e0187394. doi: 10.1371/journal.pone.0187394 (PMC5718422; doi:10.1371/journal.pone.0187394)
Supplement: S1 Appendix — (DOCX) [file pone.0187394.s001.docx]

**S1 Appendix: Statistical methods, surveys, and additional results.**

Three different types of dependent variables are used in this study and each requires its own statistical technique. The addition of honorary authors to manuscripts and to grant proposals has a categorical dependent variable set equal to one if an individual added an author to a grant or felt an obligation to add authors to a manuscript, and was equal to zero otherwise. With a binary dependent variable, OLS estimation produces heteroscedastic errors and can lead to predicted probabilities outside the [0, 1] interval. To counter these issues, we estimate a logit model, which transforms the dependent variable by taking the natural log of the odds ratio (p/(1-p)) where p = the probability of the event. Thus, the probability of an occurrence (adding an honorary author) or y = 1, given a set of predictors, *x_i_* is

Pr (*y_i_* = 1| *x_i_*) = *e^z^* / (*e^z^* + 1) where *z* = β_0_ + β_1_ *x*_1_ + β_2_ *x*_2_ + . . . + β*_k_* *x_k_*

Using data from the survey described below, we test whether honorary authorship is related to academic rank, gender, discipline, and co-authorship as hypothesized. Drilling down to explore the underlying reasons individuals add authors also involved a binary dependent variable and again logit estimation was employed.

To explore the extent and frequency of coercive citation, we use two different dependent variable constructs. The first set of tests have a binary dependent variable, whether respondents were coerced or not. This has the same non-linear characteristic as in the honorary author model and, once again, we use logit estimation. We also gather data on the frequency of coercion; asking our respondents *how many times* they had been coerced in the last five years. This is count data and the standard approach to modeling count data is Poisson regression. However, a likelihood ratio test shows that these data are over dispersed (the conditional variance being significantly larger than the conditional mean) and so we use a negative binomial regression model. This introduces an omitted parameter, *u*, to the conditional mean of a Poisson distribution and assuming the density of *u* is a gamma distribution, the distribution of *y_i_* (*y_i_* = *y + u*) is once again Poisson with conditional mean and variance. To insure non-negative predictions, a log-linear form is used so:

$$\ln y_{i}=\ln(y+u)=e^{\beta x+\varepsilon}$$

or $\ln y=\beta^{'}x+\varepsilon+\eta$,

where $\eta=\ln u$ accounts for the over dispersion.

The dependent variable used to study padded citation is different once again. Here the data consist of responses to the statements like, “I would likely add citations before submission” [for manuscripts] and “I am likely to skew my citations to high impact journals” [for grant proposals]. The responses fall into one of five categories: strongly agree, agree, neutral, disagree, and strongly disagree; multiple categories in which the order of the categories matter. To account for the categorical nature of these data and take advantage of the ordering information, we estimate this model with an ordered logit procedure. Ordered logit is a generalization of the logit model that allows for several different observed responses, *y_i_* = {1, 2, 3,..} that lie in some natural order {strongly disagree, disagree, neutral, …}. We observe *y_i_ = j* if the underlying latent variable lies between the cutpoints, $\alpha_{j-1} \text{and} \alpha_{j}$. As a respondent becomes more passionate about an issue there is a point, $\alpha_{j-1}$, beyond which his response shifts from “neutral” to “agree” and so forth. The explanatory variables in *x* are expected to influence this intensity of feeling and so the probability that the indicator variable moves from one category of coercion as a logistic function or

$$\begin{matrix} \Pr(y_{i}=j|x_{i})=\Pr(\alpha_{j-i}<x_{i}^{'}\beta+u_{i}\leq\alpha_{j}) \\ =\Pr(\alpha_{j-1}-x_{i}^{'}\beta<u_{i}\leq\alpha_{j}-x_{i}^{'}\beta) \\ =F(\alpha_{j}-x_{i}^{'}\beta)-F(\alpha_{j-i}-x_{i}^{'}\beta) \end{matrix}$$

where *F* is the cdf of the logistic distribution.

**The Surveys**

This section presents the survey questions and counts of the raw responses.

**Honorary Authorship in Manuscripts**

Some scholars feel obligated to add individuals, often senior researchers, to the list of coauthors of their manuscripts even though the contributions of those individuals are minimal. This has been called Honorary Authorship. The first few questions address this practice.

1. Prior to this survey were you aware of this practice?

Yes 9509 No 1180

1. How appropriate or how inappropriate do you view this practice?

Extremely inappropriate 3382

Inappropriate 5918

Neutral 1651

Appropriate 348

Extremely Appropriate 107

3. Have YOU felt obligated to add the name of another individual as a coauthor to your manuscript even though that individual's contribution was minimal?

Yes 3749 No 6949

4. In the last five years HOW MANY TIMES have you added or had coauthors added to your manuscripts even though they contributed little to the study?

Average = 2.41; s.d. = 4.43

5. Please focus on the most recent incidence in which an individual was added as a coauthor to one of your manuscripts even though his or her contribution was minimal.

Including yourself, how many authors were on this manuscript?

Average = 6.8; s.d. = 74.8. This includes manuscripts with up to 3500 authors.

Average = 4.0; s.d. = 3.05. This eliminates manuscripts with > 50 authors.

6. Even though this individual added little to this manuscript he (or she) was included as an author. The main reason for this inclusion was:

Director: was the Director of the lab or facility used in the research 875

Authority: occupies a position of authority and can influence my career. 658

Mentor: this is my mentor 406

Colleague: this is a colleague I wanted to help out 263

Reciprocity: I was included or expect to be included as a co-author on their work 262

Data: they had data I needed. 236

Reputation: their reputation increases the chances of the work being published 210

Funding: they had funding we could apply to the research 166

Please focus on your most recently accepted manuscript and refer to that experience when you answer this question.

7. Including yourself, how many authors were on your most recently accepted paper?

Average = 5.9; s.d. = 78.7 Including manuscripts with up to 3500 authors

Average = 3.5; s.d. = 2.61 Eliminating manuscripts with > 50 authors

**Coercive Citation and Padded Citations in Manuscripts**

Some editors ask authors who have a manuscript under review at their journal to add citations to articles in the editor's journal even though those articles are not critical to the manuscript under review. Such citation requests are not based on a perceived omission in the manuscript's academic content, these editors are simply asking authors to add citations to their home journal as a condition of acceptance or review.

1. Prior to this survey, were you aware of this practice?

Yes 4450 No 7848

1. How appropriate or how inappropriate do you view this practice (requesting citations to the editor's home journal for reasons not based on content)?

Extremely Inappropriate 7046

Inappropriate 4119

Neutral 720

Appropriate 216

Extremely Appropriate 188

1. Have YOU received a request from an editor to add citations from the editor’s journal for reasons that were not based on content?

Yes 1735 No 10572

1. In the last five years, approximately HOW MANY TIMES have you received a request from the editor to add more citations from the editor’s journal for reasons that were not based on content?

Average = 2.03; s.d. = 1.48

1. Please focus on the most recent incident in which an editor asked you to add citations not based on content. Including yourself, how many authors were on this manuscript?

Average = 3.27; s.d. = 2.1

YES, I've been asked to add citations

Please focus on your most recently accepted manuscript and refer to that experience

when you answer this question.

5a. Including yourself, many authors were on your most recently accepted paper?

Average = 7.37; s.d. = 85.9 Includes manuscripts with > 50 authors.

Average = 3.85; s.d. = 2.54 Eliminates manuscripts with > 50 authors.

How strongly do you agree or disagree with the following statements?

1. "If an editor asks authors to add citations to their home journal for reasons not based on the content of the manuscript that reduces the prestige of the journal in my eyes."

Strongly Agree 5652

Agree 4710

Neutral 1134

Disagree 452

Strongly Disagree 146

2. “All else equal, I am less likely to submit manuscripts to journals that make these requests.”

Strongly Agree 4440

Agree 4538

Neutral 2051

Disagree 885

Strongly Disagree 180

3. "If I were submitting an article to a journal with a reputation of asking for citations to itself even if those citations are not critical to the content of the article, I would probably add such citations BEFORE SUBMISSION."

Strongly Agree 2125

Agree 3061

Neutral 2025

Disagree 3892

Strongly Disagree 950

4. To track the possible spread of this practice we need to know specific journals. Would you please provide the names of journals you know engage in this practice?

1735 respondents said they have been coerced but 479 preferred not to name a journal. The remaining 1256 respondents named 611 journals. Of these, the average number of times a journal was named as a coercer was 2.05 times and s.d. = 3.60.

**Honorary Authorship and Padded Citations in Grants**

1. In the last five years approximately how many grant proposals have you submitted for funding?

Average = 4.55; s.d. = 6.55

2. Approximately how much grant money have you received in the last five years? Please write your estimated dollars in box; enter 0 if zero.

Average = $4,818,000; s.d. = $227,720,000

Average = $925,000; s.d. = $2,652,000

(The second set of statistics eliminates 17 entries reporting 100 million dollars or more.)

How strongly do you agree or disagree with the following statements?

3. Grant reviewers’ evaluations of grant proposals are influenced by citations to journals with high impact factors.

Strongly Agree 1589

Agree 4467

Neutral 2868

Disagree 997

Strongly Disagree 198

Opinion

4. When developing a grant proposal I tend to skew my citations toward high impact factor journals, even if those citations are of marginal import to my proposal.

Strongly Agree 261

Agree 1322

Neutral 2897

Disagree 3842

Strongly Disagree 1667

5. Have you ever felt obligated to add a scholar's name to a grant proposal even though you knew that individual would not make a significant contribution to the research effort?

Yes 2099 No 7973

1. The main reason you added an individual to this grant proposal even though he (or she) was not expected to make a significant contribution was:

Director: was the Director of the lab or facility used in the research 293

Authority: occupies a position of authority and can influence my career. 281

Mentor: this is my mentor 176

Colleague: this is a colleague I wanted to help out 34

Reciprocity: I was included or expect to be included as a co-author on their work 19

Data: they had data I needed. 11

Reputation: their reputation increases the chances of receiving funding 1348

Reviewers: adding author(s) was suggested by grant reviewers 55

7. To which agency, organization, or foundation was this proposal directed?

NSF 1866

HHS 1689

Corporations 165

Private nonprofit 787

State funding 446

Other Federal grants 507

Other grants 539

**General Information**

1. What is your current rank?

Assistant Professor 1878

Associate Professor 2418

Professor 4080

Research Faculty 280

Clinical Faculty 182

1. In what discipline do you do your primary research?

Medicine 2642

Nursing 874

Accounting 259

Economics 832

Finance 272

Information Systems 515

Management 717

Marketing 606

Political Science 370

Psychology 898

Sociology 470

Biology 1183

Chemistry 817

Computer Science 415

Ecology 345

Engineering 956

Mathematics 381

Physics 564

1. Within the last five years, approximately how many publications, including acceptances, do you have?

Average 12.1; s.d. = 14.78

1. Gender:

Male 7963 Female 3956

**Response rates:** As stated in the main text, the approximately 110,000 surveys yielded over 12,000 responses for an overall response rate of about 10.5%. Response rates did vary across disciplines and those rates are displayed in Table A.

**Table A: Response rates by discipline**

| Discipline | Response rates |
| --- | --- |
| Medicine | 14.7% |
| Nursing | 12.3% |
| Accounting | 9.4% |
| Economics | 14.4% |
| Finance | 7.8% |
| Information Systems | 6.4% |
| Management | 8.5% |
| Marketing | 8.2% |
| Political Science | 7.5% |
| Psychology | 12.1% |
| Sociology | 9.0% |
| Biology | 5.2% |
| Chemistry | 13.9% |
| Computer Science | 10.1% |
| Ecology | 7.1% |
| Engineering | 10.6% |
| Mathematics | 9.6% |
| Physics | 9.6% |

There does not seem to be a pattern in this variation; however, the five disciplines with the highest response rates (Medicine, Economics, Chemistry, Nursing, and Psychology) come from four different major fields (medical, business, science, and social science) and disciplines from three of those four majors fields (business, science, and social science) also have some of the lowest response rates (information systems, biology, and political science). Calculating a rank correlation coefficient between the response rate by discipline and the estimated coefficients for honorary authors from Table 3 yields *r_s_* = -0.181; these are not correlated.

**Sample versus population:** Any survey study has to address the question of representativeness; that is, do the responses represent the broader population. A common method to look for bias is to compare some attributes between the population and survey respondents. For most of the disciplines in this study, we were able to get a demographic statistic (sex) and the academic rank of the target population. We can then compare those with the respondent characteristics. That comparison appears below in Table B.

**Table B: Population versus sample demographics**

|  | **% male** | **% female** | **% Professor** | **% Associate**  **Professor** | **% Assistant**  **Professor** | **% Other** |
| --- | --- | --- | --- | --- | --- | --- |
| **Medicine** | 66.1%/55.8% | 33.9%/31.0% | 29.0%/35.8% | 20.1%/21.2% | 33.5%/17.1% | 17.5%/25.9% |
| **Nursing** | 6.4%/6.9% | 93.6%/82.0% | 15.2%/19.1% | 17.2%/23.2% | 31.7%/28.9% | 35.9%/28.8% |
| **Accounting** | 66.9%/69.6% | 33.1%/28.4% | 20.6%/37.4% | 17.2%/28.4% | 20.1%/25.8% | 42.1%/8.4% |
| **Finance** | 83.5%/82.3% | 16.5%/17.6% | 29.5%/56.2% | 17.5%/18.4% | 20.6%/25.7% | 32.4%/0% |
| **Info systems** | 76.3%/72.3% | 23.7%/23.45 | 21.1%/31.9% | 21.4%/31.9% | 13.2%/20.2% | 44.3%/16% |
| **Marketing** | 70.3%/66.7% | 29.7%/31.7% | 26.9%/40.0% | 18.8%/32.8% | 19.8%/20.5% | 34.5%/6.7% |
| **Political science** | 69.6%/69.4% | 30.4%/30.0% | 34.6%/37.3% | 23.5%/34.6% | 20.2%/18.1% | 21.2%/10.0% |
| **Psychology** | 57.0%/52.4% | 43.0%/46.0% | 35.6%/38.9% | 17.9%/23.3% | 14.4%/16.5% | 32.1%/21.3% |
| **Sociology** | 54.2%/52.8% | 45.3%/45.5% | 35.5%/42.5% | 24.3%/25.3% | 16.5%/18.1% | 23.7%/14.1% |
| **Biology** | 68.9%/68.4% | 31.1%/30.3% | 39.8%/39.1% | 15.7%/24.3% | 11.4%/14.9% | 33.1%/21.7% |
| **Chemistry** | 76.2%/77.5% | 23.8%/22.5% | 43.4%/45.4% | 19.3%/25.3% | 19.3%/13.4% | 18.0%/15.8% |
| **Computer science** | 81.4%/80.5% | 18.6%/15.7% | 38.9%/47.7% | 26.5%/28.6% | 15.2%/10.4% | 19.4%/13.3% |
| **Ecology** | 72.0%/69.0% | 28.0%/28.7% | 34.0%/40.3% | 16.4%/16.8% | 12.6%/10.7% | 37.0%/32.3% |
| **Engineer** | 87.7%/82.5% | 12.3%/16.2% | 52.2%/50.7% | 18.6%/20.5% | 15.3%/11.0% | 13.9%/17.8% |
| **Mathematics** | 74.7%/86.4% | 25.35/17.8% | 40.1%/45.3% | 18.4%/27.5% | 14.9%/10.7% | 26.6%/16.5% |
| **Physics** | 88.0%/87.7% | 12.0%/10.5% | 50.7%/53.9% | 14.4%/15.3% | 12.5%/7.0% | 22.4%/23.8% |

The first number in each cell represents the percentage of individuals in the population with that cell’s demographic characteristic and the second number represents the percentage of the sample’s population with that characteristic.

The results are encouraging because in the vast majority of cases the respondent distribution of characteristics follows the population. The male/female split is quite close. Academic ranks show greater differences between the population and sample, but that difference is primarily due to the fact that we had more missing observations of academic rank in the target population than from our respondents (see the last column in Table B). Recognizing that difference, there is no compelling reason to suspect that our survey is biased, but of course it ultimately remains as an unknown.

The most intuitive concern would be if individuals who had experience with manipulation were significantly more likely (or less likely) to answer the survey because that could inflate (or deflate) some of our results. However, if that was the case then we would expect to see at least a moderately strong and positive rank correlation coefficient between the likelihood of manipulating and response rates. That is, disciplines with more manipulation would have a higher portion of their populations experiencing manipulation and therefore would have a higher portion responding. There is no evidence of this. The rank correlation coefficient calculated above (*r_s_* = -0.181) suggests virtually no correlation. And finally, even if our sample is biased towards individuals who have more experience with manipulation, the sheer numbers of responses that report manipulation suggests that manipulation is not a minor problem.

**Additional Results**

**Honorary authors, standardized coefficients:** As discussed in the main text, one can get additional information of the effect size across different, independent variables by standardizing the continuous variables and re-estimating. In this case, the estimated coefficients represent the impact of a one standard deviation change in the independent variable on the dependent variable. The standardized estimates for the honorary authorship models in Table 3 and Table 4 are given below in Table C and Table D.

**Table C: Adding honorary authors to manuscripts: Estimate coefficients and odds ratios; standardized coefficients**

| Variables | Estimated coefficients | Std. error | Odds ratio | | | Std. error | | |
| --- | --- | --- | --- | --- | --- | --- | --- | --- |
| Academic Ranks | | | | | | | | |
| Assistant Professor | 0.642** | 0.061 | | | 1.901** | | 0.117 | |
| Associate Professor | 0.341** | 0.056 | | | 1.407** | | 0.079 | |
| Lecturer | 0.085 | 0.137 | | | 1.089 | | 0.150 | |
| Research Faculty | 0.716** | 0.129 | | | 2.046** | | 0.265 | |
| Clinical Faculty | 0.411* | 0.169 | | | 1.508* | | 0.255 | |
| Other rank | 0.504** | 0.127 | | | 1.655** | | 0.211 | |
| Gender and number of co-authors | | | | | | | | |
| Male | -0.471** | 0.050 | | | 0.624** | | 0.031 | |
| # co-authors (std) | 0.096** | 0.023 | | | 1.10** | | 0.025 | |
| Disciplines | | | | | | | | |
| Medicine | 0.191** | 0.055 | | 1.211** | | | | 0.068 |
| Nursing | 0.148 | 0.084 | | 1.161 | | | | 0.098 |
| Accounting | -0.615** | 0.200 | | 0.541** | | | | 0.108 |
| Economics | -0.218* | 0.094 | | 0.804* | | | | 0.075 |
| Finance | -0.105 | 0.195 | | 0.900 | | | | 0.175 |
| Info systems | 0.377 | 0.209 | | 1.458* | | | | 0.305 |
| Management | 0.491** | 0.089 | | 1.634** | | | | 0.146 |
| Marketing | 0.561** | 0.149 | | 1.752** | | | | 0.262 |
| Political Sci | -0.819** | 0.141 | | 0.441** | | | | 0.062 |
| Psychology | 0.056 | 0.076 | | 1.058 | | | | 0.080 |
| Sociology | 0.052 | 0.101 | | 1.054 | | | | 0.107 |
| Biology | 0.123 | 0.068 | | 1.131** | | | | 0.077 |
| Chemistry | -0.352** | 0.103 | | 0.703** | | | | 0.073 |
| Computer Sci | 0.040 | 0.131 | | 1.041 | | | | 0.136 |
| Ecology | 0.300** | 0.113 | | 1.349** | | | | 0.153 |
| Engineer | 0.145 | 0.088 | | 1.156 | | | | 0.101 |
| Mathematics | -0.527** | 0.170 | | 0.590** | | | | 0.100 |
| Physics | 0.151 | 0.110 | | 1.163 | | | | 0.128 |
| Publication history | | | | | | | | |
| Publications (std) | 0.064** | 0.007 | | 1.066** | | 0.008 | | |
| Constant | -0.986** | 0.063 | | 0.373** | | 0.023 | | |
|  | n = 9910**;** 𝛘^2^= 524.11 | | | | | | | |

Logit regression, dependent variable is binary: 1 = added author, 0 = did not add author to research. Independent variables include academic ranks, disciplines, gender, number of co-authors, and the number of publications. * Indicates significance at the 5% level; ** significant at the 1% level.

**Table D: Number of times authors added to manuscripts: Estimated coefficients**

**and incidence rate ratios; standardized coefficients**

|  | **Estimated coefficient** | **Standard Error** |  | **Incidence rate ratio** | **Standard error** |
| --- | --- | --- | --- | --- | --- |
| **Faculty Ranks** |  |  |  |  |  |
| **Assistant Professor** | 0.658** | 0.059 |  | 1.931** | 0.113 |
| **Associate Professor** | 0.343** | 0.054 |  | 1.409** | 0.076 |
| **Lecturer** | 0.147 | 0.135 |  | 1.159 | 0.157 |
| **Research Faculty** | 0.801** | 0.123 |  | 2.227** | 0.274 |
| **Clinical Fac.** | 0.175 | 0.173 |  | 1.192 | 0.206 |
| **Other rank** | 0.501** | 0.122 |  | 1.650** | 0.201 |
| **Gender and number of co-authors** | | | | | |
| **Male** | -0.266** | 0.049 |  | 0.766** | 0.037 |
| **Number of co-authors (std)** | 0.234** | 0.027 |  | 1.264** | 0.034 |
| **Disciplines** |  |  |  |  |  |
| **Medicine** | 0.138** | 0.054 |  | 1.148** | 0.062 |
| **Nursing** | 0.201* | 0.083 |  | 1.223** | 0.102 |
| **Accounting** | -0.650** | 0.199 |  | 0.552** | 0.104 |
| **Economics** | -0.072 | 0.089 |  | 0.930 | 0.083 |
| **Finance** | -0.070 | 0.189 |  | 0.932 | 0.176 |
| **Info systems** | 0.254 | 0.209 |  | 1.289 | 0.269 |
| **Management** | 0.515** | 0.087 |  | 1.674** | 0.146 |
| **Marketing** | 0.398** | 0.150 |  | 1.488** | 0.222 |
| **Political Sci** | -0.718** | 0.134 |  | 0.487** | 0.065 |
| **Psychology** | 0.044 | 0.074 |  | 0.957 | 0.071 |
| **Sociology** | 0.010 | 0.101 |  | 0.990 | 0.100 |
| **Biology** | 0.149* | 0.066 |  | 1.161* | 0.076 |
| **Chemistry** | -0.587** | 0.104 |  | 0.555** | 0.058 |
| **Computer Science** | 0.111 | 0.126 |  | 1.118 | 0.141 |
| **Ecology** | 0.325** | 0.109 |  | 1.383** | 0.150 |
| **Engineering** | 0.299** | 0.083 |  | 1.348** | 0.112 |
| **Mathematics** | -0.317* | 0.154 |  | 0.728* | 0.112 |
| **Physics** | 0.078 | 0.107 |  | 1.081 | 0.115 |
| **Other controls** |  |  |  |  |  |
| **Publications (std)** | 0.114** | 0.008 |  | 1.120** | 0.009 |
| **Constant** | -1.086** | 0.063 |  | 0.337** | 0.021 |
|  | n = 9929; 𝛘^2^= 731.5 | | | | |

Negative binomial regression, dependent variable is the number of times the respondent added honorary authors to manuscripts in the last five years. * Indicates significance at the 5% level; ** significant at the 1% level.

The standardized coefficients suggest that in both models a standard deviation in the number of authors has a larger impact than a standard deviation increase in the number of publications.

**Reasons authors are added:** Table 6 of the main text displays the estimated coefficients and standard errors of the logit estimation for reason authors added an honorary author to their manuscript. Table E given below presents the odds ratios for those estimates.

**Table E: Reasons authors added to manuscripts: Odds ratios**

|  | **Director of Laboratory** | | | | | **Position of Authority** | | | **Mentor** | | |
| --- | --- | --- | --- | --- | --- | --- | --- | --- | --- | --- | --- |
|  | | Odds  Ratio | | Std. err | | Odds  Ratio | | Std. err | Odds  Ratio | | Std. err |
| **Academic Ranks** | |  | |  | |  | |  |  | |  |
| **Assistant Professor** | | -0.996 | | 0.115 | | 1.592** | | 0.204 | 2.523** | | 0.406 |
| **Associate Professor** | | 1.048 | | 0.115 | | 1.467** | | 0.185 | 1.231 | | 0.217 |
| **Lecturer** | | 0.691 | | 0.229 | | 1.454 | | 0.434 | 3.948** | | 1.149 |
| **Research Faculty** | | 1.512 | | 0.320 | | 1.628* | | 0.408 | 1.714 | | 0.556 |
| **Clinical Faculty** | | 1.421 | | 0.439 | | 0.688 | | 0.256 | 3.153** | | 1.095 |
| **Other Rank** | | 1.383 | | 0.329 | | 1.283 | | 0.343 | 1.629 | | 0.495 |
| **Gender and co-authorship** | | | | | | | | | | | |
| **Male** | | | 1.087 | | 0.102 | | 0.928 | 0.098 | | 0.973 | 0.130 |
| **Number of Co-authors** | | | 1.004 | | 0.015 | | 0.965 | 0.020 | | 0.807** | 0.029 |
| **Disciplines** | | |  | |  | |  |  | |  |  |
| **Medicine** | | | 2.370** | | 0.296 | | 1.678** | 0.195 | | 1.454* | 0.243 |
| **Nursing** | | | 1.449* | | 0.271 | | 2.112** | 0.343 | | 2.330** | 0.466 |
| **Accounting** | | | 0.403 | | 0.282 | | 1.459 | 0.611 | | 0.561 | 0.394 |
| **Economics** | | | 0.626 | | 0.162 | | 1.069 | 0.227 | | 1.554 | 0.370 |
| **Finance** | | | 0.195 | | 0.188 | | 1.244 | 0.549 | | 0.848 | 0.503 |
| **Info systems** | | | 1.116 | | 0.484 | | 1.387 | 0.540 | | 2.806** | 1.097 |
| **Management** | | | 0.709 | | 0.155 | | 1.662** | 0.268 | | 1.997** | 0.379 |
| **Marketing** | | | 0.765 | | 0.268 | | 0.949 | 0.293 | | 1.717* | 0.555 |
| **Political Science** | | | 0.416 | | 0.207 | | 1.292 | 0.410 | | 0.486 | 0.278 |
| **Psychology** | | | 2.340** | | 0.360 | | 0.721 | 0.131 | | 1.403 | 0.290 |
| **Sociology** | | | 1.105 | | 0.257 | | 0.797 | 0.192 | | 0.600 | 0.207 |
| **Biology** | | | 2.614** | | 0.370 | | 0.484** | 0.094 | | 0.635 | 0.162 |
| **Chemistry** | | | 2.359** | | 0.503 | | 0.400** | 0.142 | | 0.604 | 0.251 |
| **Computer Science** | | | 0.399* | | 0.164 | | 1.481 | 0.392 | | 1.097 | 0.410 |
| **Ecology** | | | 2.001** | | 0.415 | | 0.901 | 0.228 | | 1.377 | 0.431 |
| **Engineering** | | | 2.096** | | 0.360 | | 0.782 | 0.164 | | 0.688 | 0.209 |
| **Mathematics** | | | 0.796 | | 0.368 | | 0.617 | 0.312 | | 0.273 | 0.264 |
| **Physics** | | | 1.105 | | 0.268 | | 0.717 | 0.208 | | 0.928 | 0.362 |
| **Publication history** | | |  | |  | |  |  | |  |  |
| **Publications** | | | 1.005 | | 0.003 | | 0.994 | 0.004 | | 0.995 | 0.006 |
| **Constant** | | | 0.164** | | 0.023 | | 0.194** | 0.028 | | 0.141** | 0.288 |
|  | | | n = 3158; 𝛘^2^ =138.1 | | | | n = 3158; 𝛘^2^ = 136.4 | | | n = 3158; 𝛘^2^ = 192.75 | |

Logit regression, dependent variable is binary: 1 = added director of laboratory as co-author, or someone in position of authority, or a mentor (even though they were not materially involved in the research),

0 = some other reason for adding author. Independent variables include academic ranks, disciplines, gender, number of co-authors, and number of publications in last 5 years. * Indicates significance

at the 5% level; ** significant at the 1% level.

Table 7 in the main text gives the estimated coefficients and standard errors for the models exploring reasons individuals added honorary authors to grant proposals. The transformed odds ratios for those estimates are given below in Table F.

**Table F: Reasons authors are added to grant proposals: Odds ratios**

| **Variables** | **Added author** | | **Reputation** | | **Director** | | **Authority** | | | |
| --- | --- | --- | --- | --- | --- | --- | --- | --- | --- | --- |
|  | Odds  Ratios | Std. err | Odds  Ratios | Std. err | Odds  Ratios | Std.  err | | Odds  Ratios | | Std. err |
| **Academic ranks and Gender** | | | | | | | | | | |
| **Assistant** | 1.72** | 0.15 | 1.36* | 0.19 | 0.63* | 0.13 | 1.22 | | | 0.27 |
| **Associate** | 1.59** | 0.13 | 1.25 | 0.16 | 0.76 | 0.15 | 1.30 | | | 0.27 |
| **Lecturer** | 2.63** | 0.60 | 1.43 | 0.53 | 0.29 | 0.22 | 1.26 | | | 0.72 |
| **Res. faculty** | 2.38** | 0.39 | 0.86 | 0.20 | 1.98* | 0.56 | 0.80 | | | 0.34 |
| **Clinic faculty** | 0.88 | 0.34 | 0.55 | 0.29 | 1.10 | 0.74 | 2.78 | | 1.89 | |
| **Other rank** | 2.48** | 0.47 | 1.27 | 0.40 | 0.92 | 0.39 | 1.59 | | 0.68 | |
| **Male** | 0.74** | 0.05 | 1.07 | 0.12 | 0.94 | 0.15 | 0.89 | | 0.15 | |
| **Disciplines** |  |  |  |  |  |  |  | |  | |
| **Medicine** | 3.95** | 0.37 | 1.32 | 0.19 | 1.04 | 0.22 | 0.94 | | 0.22 | |
| **Accounting** | 1.49 | 0.42 | 1.00 | 0.50 | 0.36 | 0.35 | 0.49 | | 0.48 | |
| **Economics** | 1.40** | 0.19 | 1.19 | 0.28 | 0.97 | 0.33 | 0.51 | | 0.22 | |
| **Finance** | 1.28 | 0.44 | 0.62 | 0.38 | 1.56 | 1.20 | 1.99 | | 1.54 | |
| **Info systems** | 0.90 | 0.30 | 1.04 | 0.63 | 1.43 | 1.09 | 2.75 | | 1.86 | |
| **Management** | 1.70** | 0.23 | 2.08** | 0.51 | 0.50 | 0.20 | 0.86 | | 0.32 | |
| **Marketing** | 1.10 | 0.31 | 0.85 | 0.48 | 5.59** | 2.99 | 0.45 | | 0.45 | |
| **Poly science** | 0.93** | 0.12 | 1.80 | 0.73 | 0.86 | 0.51 | 1.14 | | 0.61 | |
| **Psychology** | 0.93 | 0.10 | 1.06 | 0.20 | 1.40 | 0.37 | 0.60 | | 0.20 | |
| **Sociology** | 0.94 | 0.13 | 0.98 | 0.26 | 0.56 | 0.26 | 0.64 | | 0.30 | |
| **Biology** | 0.50** | 0.05 | 0.79 | 0.15 | 0.99 | 0.29 | 1.11 | | 0.32 | |
| **Chemistry** | 0.59** | 0.83 | 0.73 | 0.19 | 2.02* | 0.64 | 0.94 | | 0.39 | |
| **Comp Science** | 0.99 | 0.17 | 1.05 | 0.33 | 0.43 | 0.25 | 1.18 | | 0.50 | |
| **Ecology** | 0.78 | 0.12 | 0.74 | 0.21 | 1.04 | 0.41 | 1.15 | | 0.46 | |
| **Engineer** | 1.18 | 0.13 | 1.02 | 0.20 | 0.97 | 0.28 | 1.23 | | 0.33 | |
| **Mathematics** | 0.59* | 0.14 | 0.737 | 0.34 | Omitted | | 1.91 | | 1.06 | |
| **Physics** | 0.71* | 0.11 | 0.877 | 0.26 | 0.79 | 0.34 | 1.36 | | 0.52 | |
| **Grant history** |  |  |  |  |  |  |  | |  | |
| **Number Grants** | 1.03** | 0.01 | 1.01 | 0.01 | -0.97 | 0.01 | 0.99 | | 0.01 | |
| **Grant dollars** | 1.00 | 2.3E-9 | 1.00 | 4.7E-9 | 0.99 | 1.E-9 | 1.00 | | 1.3E-8 | |
| **NSF** | 1.72** | 0.19 | 0.909 | 0.19 | 1.63 | 0.54 | 1.11 | | 0.34 | |
| **HHS** | 3.18** | 0.37 | 1.88** | 0.40 | 0.92 | 0.31 | 0.55 | | 0.18 | |
| **Corporation $** | 1.56* | 0.32 | 0.40** | 0.13 | 3.46** | 1.53 | 1.47 | | 0.66 | |
| **Nonprofit** | 1.03 | 0.14 | 0.91 | 0.22 | 1.86 | 0.70 | 0.79 | | 0.30 | |
| **State funding** | 1.30 | 0.20 | 0.97 | 0.26 | 1.79 | 0.72 | 0.53 | | 0.25 | |
| **Otr.FED Grants** | 1.87** | 0.27 | 0.81 | 0.21 | 1.50 | 0.59 | 1.39 | | 0.50 | |
| **Constant** | 0.12** | 0.01 | 0.82 | 0.18 | 0.15** | 0.05 | 0.14 | | 0.05 | |
|  | n=6343;  𝛘^2^= 893.4 | | n=1711; 𝛘^2^=109.0 | | n=1693;  𝛘^2^= 70.6 | | n=1711;  𝛘^2^=44.6 | | | |

Logit regression, dependent variable is binary: 1 = added director of laboratory as co-author, or someone in position of authority, or a mentor (even though they were not materially involved in the research),

0 = some other reason for adding author. Independent variables include academic ranks, disciplines, gender, number of co-authors, and number of publications in last 5 years. * Indicates significance

at the 5% level; ** significant at the 1% level.

**Coercive citation:** As discussed above and in the main test, one can get additional information of the effect size across different, independent variables by standardizing the continuous variables and re-estimating. In this case, the estimated coefficients represent the impact of a one standard deviation change in the independent variable on the dependent variable. The two continuous variables of interest here are the number of coauthors and the number of publications. Table G and Table H display these estimates.

**Table G:** **Existence of coercive citation: Estimated coefficients and odds ratios; standardized coefficients**

| Variables | Estimated  coefficient | | Std. error | Odds ratio | Std. error |
| --- | --- | --- | --- | --- | --- |
| Academic Ranks |  | |  |  |  |
| Assistant professor | 0.357** | | 0.076 | 1.429** | 0.109 |
| Associate professor | 0.195** | | 0.073 | 1.215** | 0.089 |
| Lecturer | -0.538* | | 0.212 | 0.584* | 0.124 |
| Other faculty | 0.051 | | 0.117 | 1.052 | 0.123 |
| Gender and number of coauthors | | | | | |
| Male | | 0.164* | 0.068 | 1.178* | 0.080 |
| Number coauthors (std) | | -0.175** | 0.032 | 0.839** | 0.027 |
| Disciplines | |  |  |  |  |
| Medicine | | -0.493** | 0.089 | 0.610** | 0.055 |
| Nursing | | -0.524** | 0.153 | 0.592** | 0.090 |
| Accounting | | 0.535** | 0.157 | 1.708** | 0.268 |
| Economics | | 0.235* | 0.102 | 1.265* | 0.129 |
| Finance | | 1.281** | 0.131 | 3.601** | 0.472 |
| Info systems | | 1.306** | 0.099 | 3.691** | 0.364 |
| Management | | 1.166** | 0.088 | 3.208** | 0.281 |
| Marketing | | 1.364** | 0.093 | 3.911** | 0.362 |
| Political science | | -0.942** | 0.235 | 0.390** | 0.091 |
| Psychology | | -0.621** | 0.116 | 0.537** | 0.062 |
| Sociology | | -0.377* | 0.138 | 0.686** | 0.094 |
| Biology | | -0.114 | 0.198 | 0.892 | 0.177 |
| Chemistry | | -0.886** | 0.154 | 0.412** | 0.063 |
| Computer science | | -0.448* | 0.173 | 0.639** | 0.111 |
| Ecology | | 0.778** | 0.158 | 2.178** | 0.344 |
| Engineering | | 0.582** | 0.090 | 1.789** | 0.160 |
| Mathematics | | -1.625** | 0.321 | 0.197** | 0.063 |
| Physics | | -1.215** | 0.225 | 0.297** | 0.067 |
| Publication history | |  |  |  |  |
| Publications (std) | | 0.408** | 0.029 | 1.50** | 0.043 |
| Constant | | -2.190** | 0.099 | 0.112 | 0.011** |
|  | | n = 11567; 𝛘^2^ = 1022.9 | | | |

Logit regression, dependent variable for existence of coercion is binary: 1 = have been coerced to add citations, 0 = have not been coerced; dependent variable for incidence of coercion is number of times coerced for citations in last 5 years. Independent variables include academic ranks, disciplines, gender, number of co-authors, and number of publications in last 5 years. * Indicates significance at the 5% level; ** significant at the 1% level.

**Table H:** **Frequency of coercive citation: Estimated coefficients and incidence rate ratios; standardized coefficients**

| Variables | Estimated  coefficient | | Std. error | Incidence  Rate Ratio | Std. error |
| --- | --- | --- | --- | --- | --- |
| Academic Ranks |  | |  |  |  |
| Assistant professor | 0.281** | | 0.074 | 1.324** | 0.097 |
| Associate professor | 0.094 | | 0.070 | 1.099 | 0.077 |
| Lecturer | -0.148 | | 0.179 | 0.862 | 0.155 |
| Other faculty | 0.055 | | 0.114 | 1.056 | 0.120 |
| Gender and number of coauthors | | | | | |
| Male | | 0.067 | 0.064 | 1.070 | 0.069 |
| Number coauthors (std) | | -0.067* | 0.032 | 0.935* | 0.030 |
| Disciplines | |  |  |  |  |
| Medicine | | 1.946** | 0.135 | 7.004** | 0.948 |
| Nursing | | 1.839** | 0.252 | 6.287** | 1.584 |
| Accounting | | 0.233 | 0.149 | 1.262 | 0.188 |
| Economics | | 0.051 | 0.094 | 1.052 | 0.099 |
| Finance | | 0.987** | 0.125 | 2.684** | 0.336 |
| Info systems | | 0.930** | 0.094 | 2.535** | 0.239 |
| Management | | 0.882** | 0.083 | 2.415** | 0.200 |
| Marketing | | 1.015** | 0.088 | 2.760** | 0.242 |
| Political science | | -1.115** | 0.205 | 0.328** | 0.067 |
| Psychology | | -0.848** | 0.101 | 0.428** | 0.043 |
| Sociology | | -0.590** | 0.123 | 0.554** | 0.068 |
| Biology | | -0.477** | 0.175 | 0.621** | 0.109 |
| Chemistry | | -0.923** | 0.121 | 0.397** | 0.048 |
| Computer science | | -0.736** | 0.152 | 0.479** | 0.073 |
| Ecology | | 0.211 | 0.151 | 1.235 | 0.187 |
| Engineering | | 0.280** | 0.082 | 1.323** | 0.108 |
| Mathematics | | -2.010** | 0.274 | 0.134** | 0.037 |
| Physics | | -1.676** | 0.200 | 0.187** | 0.037 |
| Publication history | |  |  |  |  |
| Total publications (std) | | 0.470** | 0.034 | 1.600** | 0.055 |
| Constant | | -1.659** | 0.098 | 0.190** | 0.019 |
|  | | n = 8951; 𝛘^2^ = 1071.1 | | | |

Logit regression, dependent variable for existence of coercion is binary: 1 = have been coerced to add citations, 0 = have not been coerced; dependent variable for incidence of coercion is number of times coerced for citations in last 5 years. Independent variables include academic ranks, disciplines, gender, number of co-authors, and number of publications in last 5 years. * Indicates significance at the 5% level; ** significant at the 1% level.

In both cases the standardized coefficient indicates a larger impact. Comparing the results from Table 8 to Table G, adding an additional author to a manuscript decreases the likelihood of being coerced by 9.2%, but increasing the number of authors by 1 standard deviation (about 2.48 authors) decreases the likelihood of coercion by 16.1%. Similarly with publications, one additional publication raises the likelihood of being coerced by 2.8% while an increase of publications by one standard deviation increases the likelihood of coercion by 50%; a much larger increase than authorship; however, a one standard deviation increase in publication equals 14.8 additional publications.
